# Deprenyl reduces inflammation during acute SIV infection

**DOI:** 10.1016/j.isci.2022.104207

**Published:** 2022-04-06

**Authors:** K.M. Emanuel, K. Runner, Z.D. Brodnik, B.M. Morsey, B.G. Lamberty, H.S. Johnson, A. Acharya, S.N. Byrareddy, R.A. España, H.S. Fox, P.J. Gaskill

**Affiliations:** 1 Department of Neurological Sciences, University of Nebraska Medical Center, Omaha, NE 68198, USA; 2 Department of Pharmacology and Physiology, Drexel University College of Medicine, Philadelphia, PA 19102, USA; 3 Department of Neurobiology and Anatomy, Drexel University College of Medicine, Philadelphia, PA 19129, USA; 4 Center on Compulsive Behaviors, NIH Intramural Research Program, Baltimore, MD 21224, USA; 5 Integrative Neuroscience Research Branch, Neuronal Networks Section, Baltimore, MD 21224, USA; 6 Department of Pharmacology and Experimental Neuroscience, University of Nebraska Medical Center, Omaha, NE 68198, USA

**Keywords:** Biological sciences, Immunology, Microbiology

## Abstract

In the era of antiretroviral therapy, inflammation is a central factor in numerous HIV-associated comorbidities, such as cardiovascular disease, cognitive impairment, and neuropsychiatric disorders. This highlights the value of developing therapeutics that both reduce HIV-associated inflammation and treat associated comorbidities. Previous research on monoamine oxidase inhibitors (MAOIs) suggests this class of drugs has anti-inflammatory properties in addition to neuropsychiatric effects. Therefore, we examined the impact of deprenyl, an MAOI, on SIV-associated inflammation during acute SIV infection using the rhesus macaque model of HIV infection. Our results show deprenyl decreased both peripheral and CNS inflammation but had no effect on viral load in either the periphery or CNS. These data show that the MAOI deprenyl may have broad anti-inflammatory effects when given during the acute stage of SIV infection, suggesting more research into the anti-inflammatory effects of this drug could result in a beneficial adjuvant for antiretroviral therapy.

## Highlights

• Deprenyl reduces peripheral and CNS inflammation in acute SIV infection
• Acute SIV infection alters monoamine levels in CNS
• Describes regional monoamine and metabolite levels in rhesus macaque brains
• Details levels of inflammatory analytes in healthy rhesus macaque peripheral blood

## Graphical abstract

**Figure:**
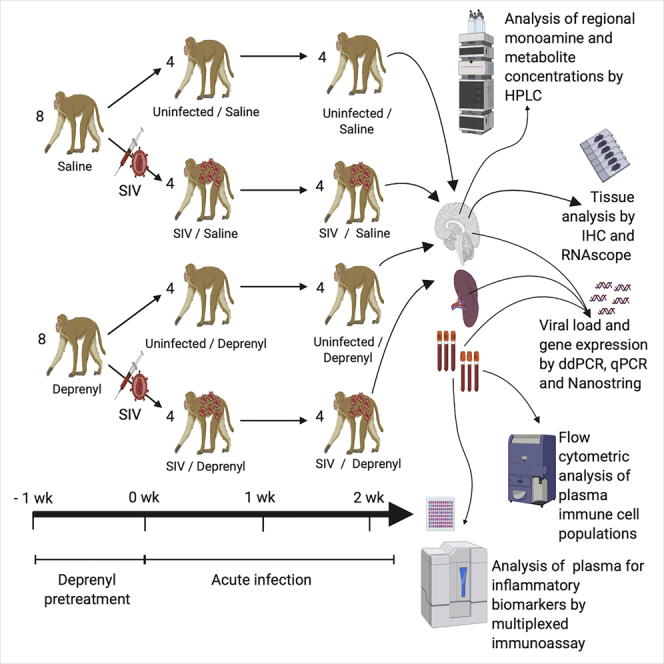

## Introduction

The HIV pandemic remains a significant public health crisis, with more than 38 million people currently infected with HIV worldwide. While there is not yet a cure for HIV, the development and use of highly efficient antiretroviral therapy (ART) has dramatically reduced HIV-associated mortality, transforming HIV infection from a terminal diagnosis to a chronic condition (Deeks et al., 2013a). For individuals with access to early diagnosis and robust treatment options, lifespan can be similar to uninfected individuals (Marcus et al., 2016, 2020; May et al., 2014). Unfortunately, chronic HIV and long-term use of ART substantially increase the risk of comorbid diseases (d'Arminio Monforte et al., 2020; Ruzicka et al., 2019), meaning the quality of the added lifespan may be worse (Collins and Armstrong, 2020). These comorbidities include cardiovascular (Freiberg et al., 2013; Triant et al., 2007), liver (Perazzo and Luz, 2017) and kidney disease (Naicker et al., 2015), cognitive impairment and neuropsychiatric disorders such as depression, substance abuse, and anxiety (Cook et al., 2018; Milanini et al., 2017; Rubin and Maki, 2019; Saylor et al., 2016; Shiau et al., 2017), a number of non-AIDS defining cancers, and metabolic diseases such as diabetes (Hernandez-Romieu et al., 2017). Depending on the study, between 30%–90% of HIV-infected individuals present with at least one comorbidity, with a substantially greater disease burden in the aging population (Farahat et al., 2020; Funke et al., 2021; Gallant et al., 2017; Lorenc et al., 2014; Pourcher et al., 2020; Ruzicka et al., 2018). Indeed, older individuals comprise the fastest growing population of people living with HIV (PLWH), and the prevalence of these comorbidities is significantly greater in PLWH than in age-matched controls, suggesting that the disease burden in PLWH will continue to grow (Kong et al., 2019; Maciel et al., 2018; Pelchen-Matthews et al., 2018; Rodriguez-Penney et al., 2013; Smit et al., 2015; UNAIDS, 2013; Yang et al., 2019).

The specific etiology for these non-AIDS comorbidities varies, but many are associated with chronic inflammation, which is prevalent in ART-treated PLWH, especially in aging populations (Deeks et al., 2013b; Negin et al., 2012; Pelchen-Matthews et al., 2018; Peterson and Baker, 2019; Yang et al., 2019). While ART suppresses viral replication and reduces systemic inflammation relative to that seen in untreated HIV infection, it does not return inflammation to pre-infection levels (Sereti et al., 2017; Temu et al., 2021; Zanni et al., 2016). Serum biomarkers of chronic, low-level inflammation, such as C-reactive protein (CRP), D-dimer, IL-1β, IL-6, IL-8, CXCL10, and TNF-α, remain elevated in ART-treated PLWH despite extended viral suppression (Funderburg, 2014; Gay et al., 2011; Jennes et al., 2004; Keating et al., 2011; Regidor et al., 2011; Sereti et al., 2017; Shive et al., 2012; Temu et al., 2021; Wada et al., 2015; Yao et al., 2013). Many of these factors are produced by myeloid cells, such as monocytes and macrophages. These markers of myeloid activation are also sustained at elevated levels in infected individuals using ART (Jennes et al., 2004; Wada et al., 2015; Yao et al., 2013). Data on T cells are mixed as some studies show ART generally reduces T cell activation (Jennes et al., 2004; Korencak et al., 2019; Yao et al., 2013), while others suggest that T cells may remain in a more active state (Adland et al., 2018; Warren et al., 2019) during ART. This shows the importance of developing supplemental therapeutics that suppresses HIV-associated inflammation in the context of ART to reduce the increasing health and financial costs associated with non-AIDS-related diseases.

One method by which this concern could be addressed is through the repurposing of existing drugs that have shown anti-inflammatory activity but are not currently used for that purpose. Monoamine oxidase inhibitors (MAOIs), such as deprenyl (also called selegiline) or rasagiline, are well studied therapeutics that is widely used to treat Parkinson disease and major depressive disorder (Finberg and Rabey, 2016). Many studies have also shown that these drugs can regulate inflammation and the production of reactive oxygen species (ROS) (Bekesi et al., 2012; Bielecka et al., 2010; Lieb, 1983; Morsali et al., 2013; Nagy et al., 2018; Sanchez-Rodriguez et al., 2020; Tsao et al., 2014). A clinical trial testing the impact of the selegiline transdermal system on cognitive impairment (ACTG5090) in 128 HIV-infected individuals on stable ART found that this system is safe and well-tolerated by HIV-infected individuals with cognitive impairment (Evans et al., 2007; Schifitto et al., 2007). Changes in inflammatory biomarkers were not examined in this trial. Still, the safety data from this study, combined with the established anti-inflammatory effects of deprenyl suggest untapped potential as an anti-inflammatory therapy during HIV infection. Further, deprenyl is an effective antidepressant, providing the possibility to simultaneously treat multiple comorbidities, a welcome effect given the high prevalence of comorbid depression in PLWH (Bhatia and Munjal, 2014; Hobkirk et al., 2015; Pinheiro et al., 2016; Wang et al., 2018).

To examine the impact of deprenyl on HIV-associated inflammation, rhesus macaques were treated with deprenyl prior to and during acute SIV infection, and the changes in infection, plasma inflammatory biomarkers, and gene expression were analyzed. While deprenyl treatment did not affect peripheral or CNS viral load, it did reduce expression of inflammatory biomarkers in the plasma. It also resulted in broad decreases in inflammatory gene expression in different regions of the CNS. To assess the efficacy of deprenyl treatment, brain regions were examined for changes in dopamine levels. Treatment with deprenyl increased dopamine and decreased DOPAC in the dopamine-rich caudate and did not affect dopamine levels in other regions with less dopamine innervation such as the hippocampus and cerebellum, indicating that the drug was acting as expected. Overall, these data confirm the safety profile for deprenyl during HIV/SIV infection and suggest that deprenyl and potentially other MAOIs have substantial untapped potential as candidates for repurposing as anti-inflammatory agents during HIV infection.

## Results

### Deprenyl does not alter SIV replication

To assess the impact of deprenyl on the development of inflammation during acute infection, rhesus macaques were treated with deprenyl prior to and during acute SIV infection. Starting one week prior to SIV infection, baseline immune measurements were taken and animals were split into two groups of eight and given daily intramuscular injections of either vehicle (saline) or deprenyl (2 mg/kg), as has been used in previous studies (Czub et al., 2001, 2004). After one week, immune measurements were repeated; four animals from each group were infected with SIVmac251, while the other four were mock-infected using saline. Daily deprenyl injections continued for two weeks following infection, with blood and CSF draws and immune measurements performed on days 7 and 14 post infection. All animals were sacrificed at 14 days post infection providing samples from four animals in each experimental group: uninfected + saline, uninfected + deprenyl, SIV + saline, and SIV + deprenyl. This early timepoint was chosen because two weeks post infection is a reproducible time in terms of viremia and levels of virus in the brain and is when inflammatory molecule production peaks in the periphery and CNS, providing the most consistency between animals (Gopalakrishnan et al., 2021; Parker et al., 2001; Roberts et al., 2004).

**Figure 1:**
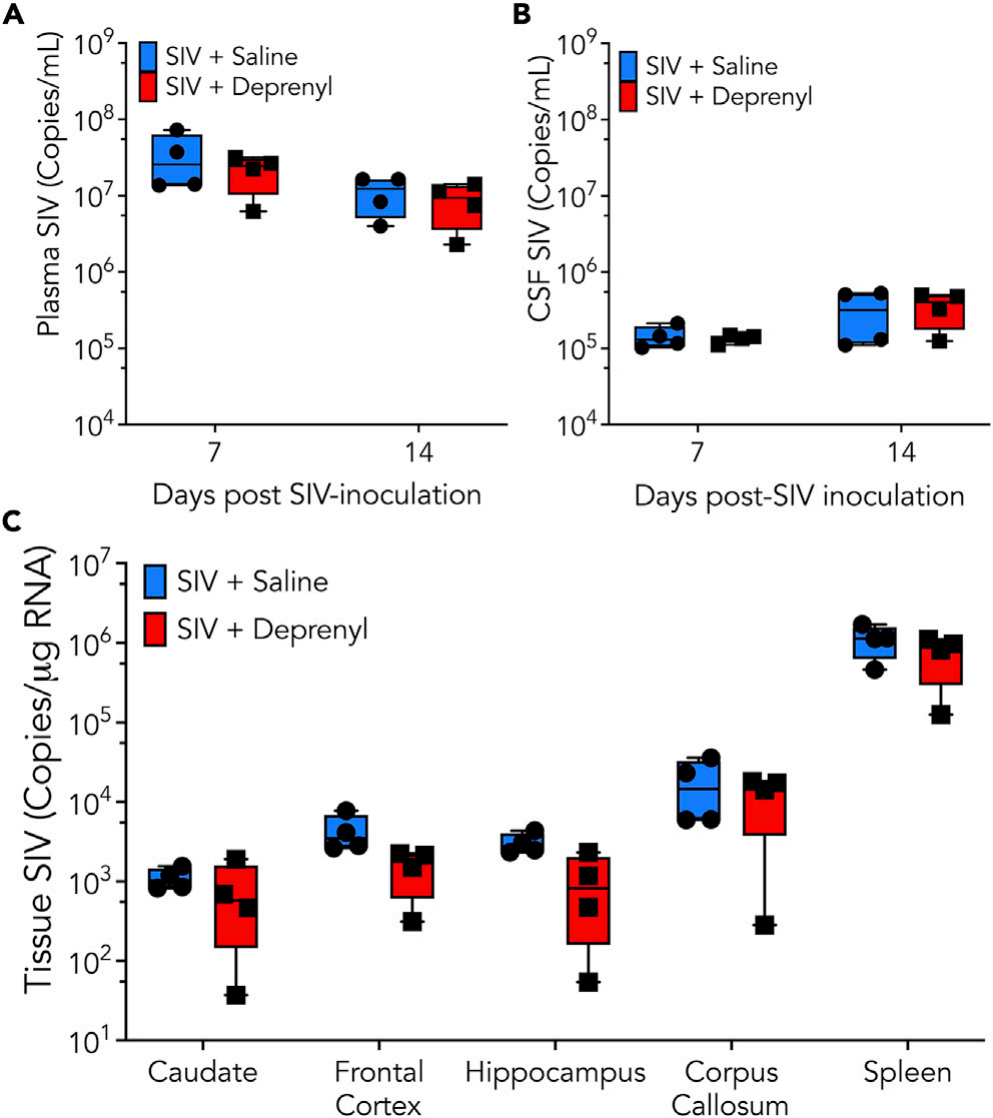
Rhesus macaques treated with either saline (blue, circles) or deprenyl (2 mg/kg, red, squares) were inoculated with SIVmac251 Both SIV + saline and SIV + deprenyl group contained four animals. At 7- and 14-day post-inoculation (dpi), plasma (A) and CSF (B) from all infected macaques were assayed for viral load (copies of SIV RNA/mL) using qRT-PCR. Tissue from frontal cortex, hippocampus, caudate, corpus callosum, and spleen isolated at 14 dpi was analyzed for copies of SIV RNA per μg of RNA using RT-ddPCR (C).

**Figure 2:**
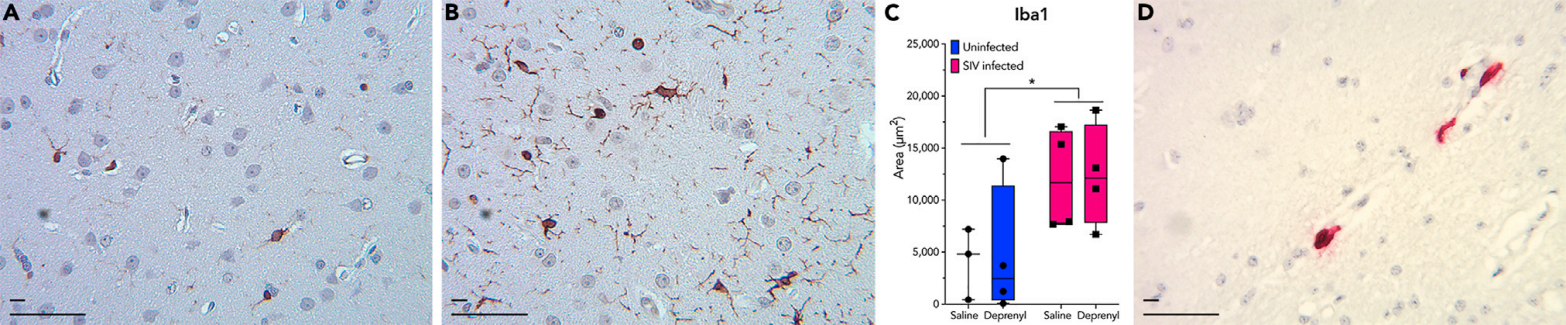
Brain tissue sections were stained with Iba-1, a common marker for activated microglia and perivascular macrophages Iba-1-positive cells were identified using immunohistochemistry in brain tissue from both uninfected (A, representative image from an uninfected macaque treated with saline) and infected (B, representative image from a SIV-infected macaque treated with saline). Deprenyl treatment did not increase the number of Iba-1-positive cells in either uninfected or SIV-infected macaques, but SIV infection significantly increased the number of Iba-1 cells [C, 2-way ANOVA SIV x deprenyl, n = 4; SIV, F (1,11) = 8.429, ∗p = 0.0144; deprenyl, F (1,11) = 0.03322, p = 0.0859]. Although SIV-infected cells could be identified by RNAscope *in situ* hybridization across the CNS (caudate shown in D), the overall numbers of infected cells were too low to quantify. Short scale bar 10 μm, long scale bar 50 μm.

To examine the impact of deprenyl on early SIV infection, we first examined the impact on viral replication. Viral load (copies of SIV RNA/mL) was quantified in the plasma and CSF in the SIV + saline and SIV + deprenyl groups at 7 and 14 days post inoculation (dpi) using qRT-PCR (Figures 1A and 1B). Several brain regions (frontal cortex, hippocampus, caudate, and corpus callosum) and the spleen were also analyzed for viral replication by assessing the copies of SIV RNA per μg of RNA at 14 dpi (Figure 1C). There was no significant difference between viral load in SIV + saline animals (blue) and SIV + deprenyl animals (red) in either biofluid or any tissue region examined. Microglial activation typifies active brain infection by HIV and SIV, and indeed a significant effect of infection was found (Figures 2A–2C). SIV-infected cells, consistent with perivascular macrophages, could be identified by *in situ* hybridization (Figure 2D); their relative rarity precluded quantification.

### Deprenyl reduces peripheral immune activity and inflammation

**Figure 3:**
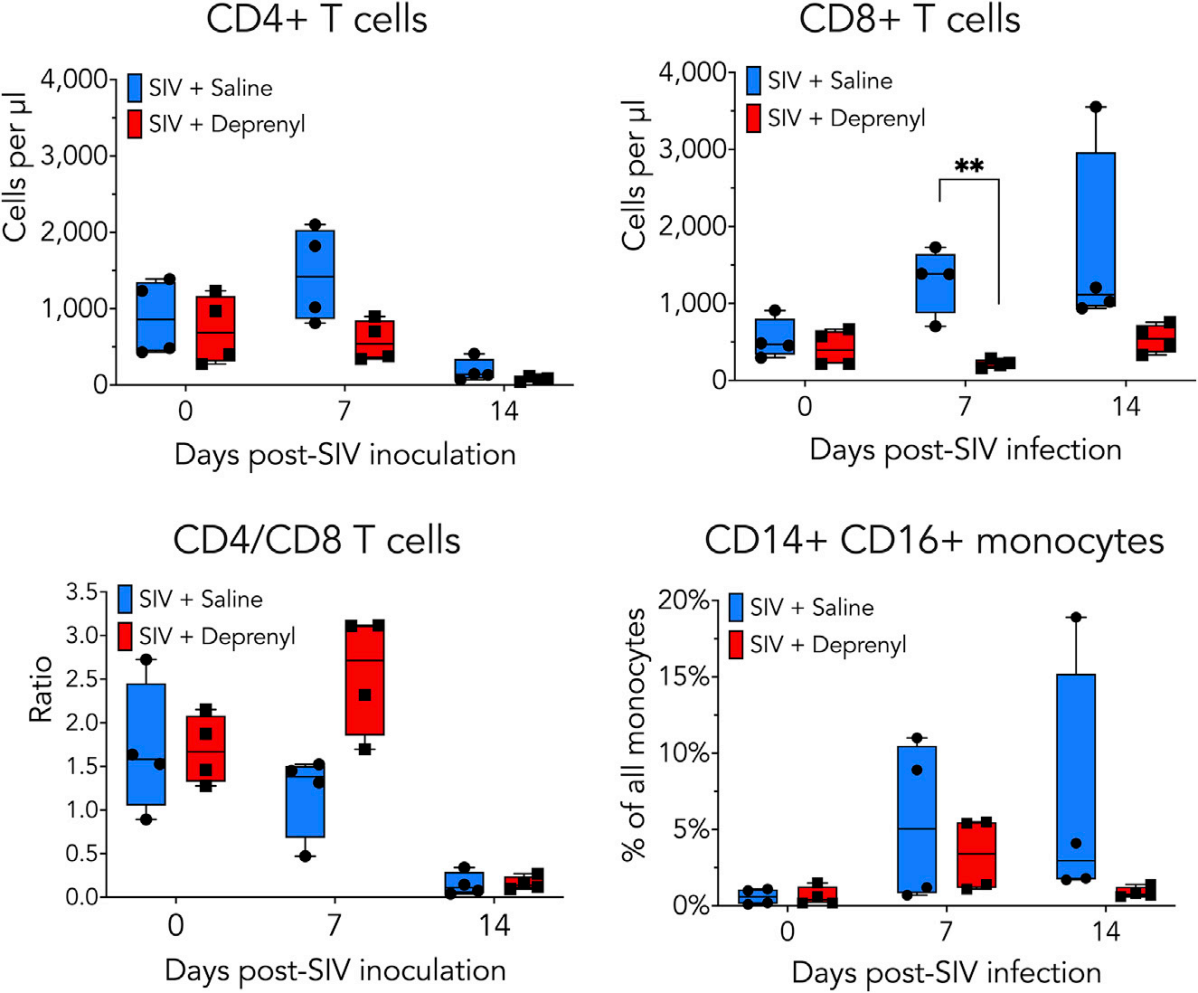
Plasma immune cell populations from SIV + saline and SIV + deprenyl macaques were evaluated by flow cytometry Flow cytometry was used to examine deprenyl-mediated changes in the numbers of CD4^+^ (A) and CD8^+^ T cells (B), the ratio of CD4 to CD8 T cells (C), as well as in the percentage of monocytes that were CD14 + CD16^+^ (D) in blood. Relative to saline-treated animals, immune populations from deprenyl-treated animals showed decreased numbers of CD4^+^ T cell and CD8^+^ T cells, and a decreased percentage of proinflammatory CD14 + CD16 ^+^ monocytes. The only significant decrease was seen in the number of CD8^+^ T cells per μL of blood [B, multiple unpaired t-tests corrected for multiple comparisons using the Holm-Sidak method, t = 4.989, ∗∗p = 0.0074]

**Figure 4:**
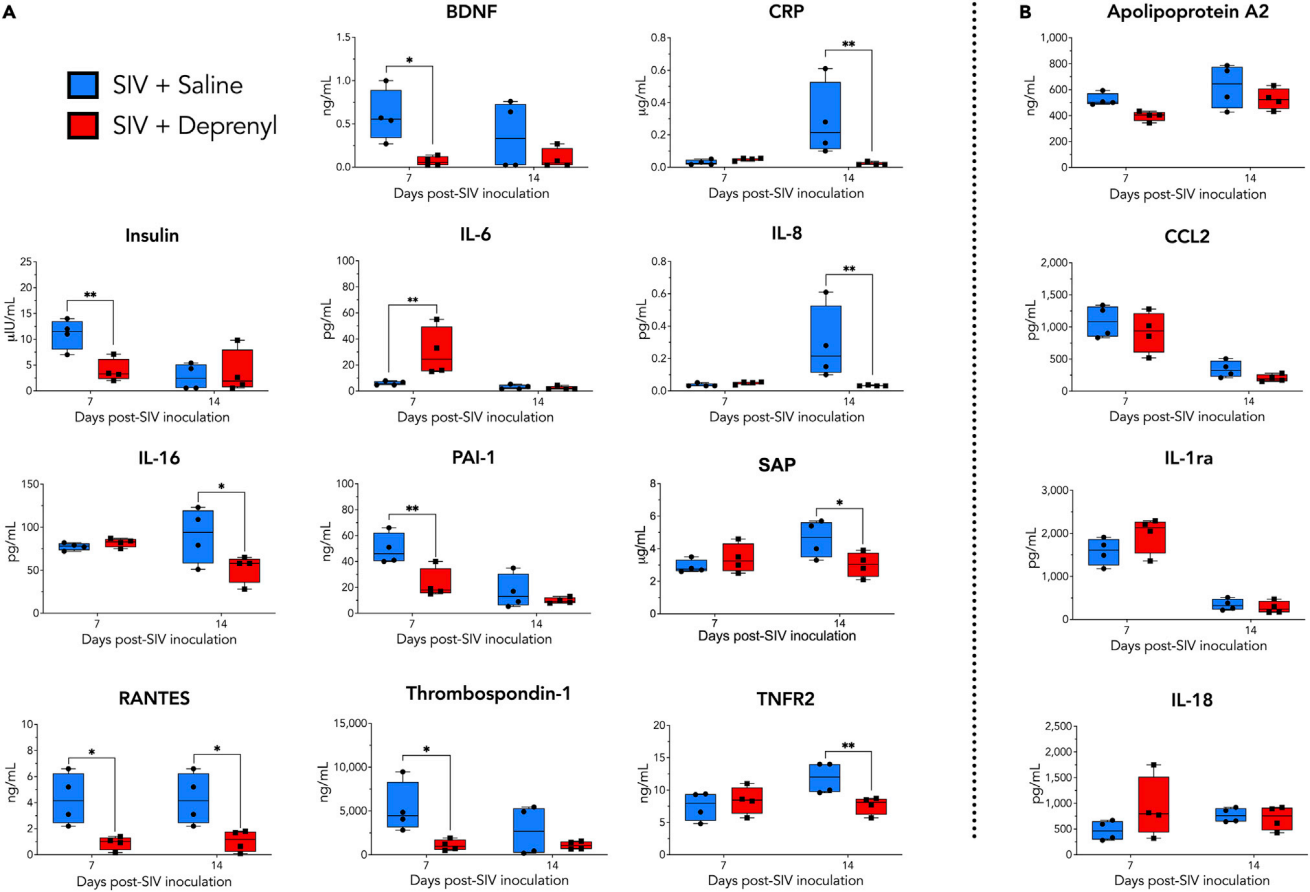
Plasma collected from all SIV-infected animals (4 SIV + saline and 4 SIV + deprenyl) was analyzed for levels of 85 distinct inflammatory factors using a Human v2.0 Multianalyte profile immunoassay (Myriad-RBM) Relative to saline-treated animals, plasma from deprenyl-treated animals showed significantly decreased concentrations of ten analytes on days 7 and/or 14 [A, multiple unpaired t-tests corrected for multiple comparisons using the Holm-Sidak method; BDNF day 7, 88.5% decrease, t = 2.905, ∗p = 0.0262; CRP day 14, 92.3% decrease, t = 3.227, ∗p = 0.0145; insulin day 7, 64.3% decrease, t = 3.244, ∗p = 0.0140; IL-8 days 14, 88.5% decrease, t = 3.106, ∗p = 0.0181; IL-6 days 7, 393.8% increase, t = 3.540, ∗∗p = 0.0081; IL-16 days 14, 42.3% decrease, t = 2.949,∗p = 0.0242; PAI-1 day 7, 54% decrease, t = 3.525, ∗∗p = 0.0084; SAP day 14, 34.2% decrease, t = 2.636, ∗p = 0.0430; CCL5 days 7 and 14; day 7 79% decrease, t = 3.194, ∗p = 0.0154; day 14, 75.2% decrease, t = 3.042, ∗p = 0.0154; thrombospondin-1 day 7, 79.5% decrease, t = 2.887, ∗p = 0.0271; TNFR2 day 14, 35.7% decrease, t = 2.887, ∗p = 0.0277]. Examples of factors not altered by deprenyl are shown (B). A total of 11 analytes were changed by deprenyl treatment in the SIV-infected animals, 35 analytes were unchanged, while another 39 analytes were not detected.

To assess the effect of deprenyl on peripheral inflammation, plasma immune populations were examined using flow cytometry and secreted immune factors were evaluated using multianalyte profiling. Immune cell populations were analyzed in plasma prior to SIV inoculation (day 0) and at 7 and 14 dpi (Figures 3A–3D). While there were changes in the immune populations between treatment groups, the small number of animals used likely limited the statistical significance of these findings. In the saline-treated animals, CD4^+^ T cells decreased, and CD8^+^ T cells increased, at 14 dpi. Animals treated with deprenyl showed decreases in CD4^+^ T cells; however, CD8^+^ T cells showed little change with infection. Relative to animals treated with saline, deprenyl-treated animals had significantly lower numbers of CD8^+^ T cells in the blood at 7 dpi. In addition, the proportion of proinflammatory monocytes (CD14 + CD16^+^) within the monocyte population was decreased in deprenyl-treated animals at 14 dpi. To measure the effect of deprenyl on secreted inflammatory factors, plasma from SIV + saline and SIV + deprenyl groups collected at day 0, 7, and 14 dpi was analyzed for levels of inflammatory factors found in the plasma (Table 1). Deprenyl significantly decreased the concentrations of ten analytes on days 7 and/or 14; brain-derived neurotrophic factor (BDNF), C-reactive protein (CRP), insulin, interleukin-8 (IL-8, CXCL8), interleukin-16 (IL-16), plasminogen activator inhibitor-1 (PAI-1), serum amyloid P-component (SAP), regulated on activation, normal T cell expressed and secreted (RANTES, CCL5), thrombospondin-1, and tumor necrosis factor receptor 2 (TNFR2). In contrast, deprenyl increased IL-6 levels at day 7. An additional 35 analytes were unchanged with deprenyl treatment. Analytes that were significantly decreased or increased (Figure 4A), as well as examples of factors not altered by deprenyl treatment (Figure 4B) are shown. The concentration and statistical comparisons of all detected analytes at 7 and 14 dpi in both SIV + saline and SIV + deprenyl groups are shown in Table 1. This table also shows the concentrations of these analytes detected in healthy rhesus macaques, none altered by deprenyl in uninfected macaques.

**Table 1:** Values of analytes detected by Human v2.0 Multianalyte profile immunoassay in SIV-infected saline versus deprenyl-treated macaques

Analyte (units)	Abbr.	Range in healthy animals	Day 7 SIV + Saline	Day 7 SIV + deprenyl	Day 7 Saline versus deprenyl	Day 14 SIV + Saline	Day 14 SIV + deprenyl	Day 14 Saline versus deprenyl
Adiponectin (ug/mL)		6.8 - 60	21.25 ± 5.32	37.25 ± 19.43	p = 0.3729	30.75 ± 13.60	42.75 ± 23.80	p = 0.3729
Alpha-1-Antitrypsin (mg/mL)	AAT	0.094 - 0.26	0.103 ± 0.012	0.128 ± 0.019	p = 0.2376	0.218 ± 0.022	0.22 ± 0.028	p = 0.8699
Alpha-2-Macroglobulin (mg/mL)	A2Macro	0.82 - 2.1	0.99 ± 0.14	1.02 ± 0.12	p = 0.7210	1.23 ± 0.15	1.3 ± 0.082	p = 0.6580
Alpha-Fetoprotein (ng/mL)	AFP	1.1-46	1.93 ± 1.85	3.18 ± 1.58	p = 0.6350	4.45 ± 2.81	9.25 ± 6.204	p = 0.1626
Apolipoprotein(a) (ug/mL)	Lp(a)	77-1130	232.5 ± 92.13	219.75 ± 100.06	p = 0.9212	467.25 ± 176.10	402.25 ± 279.226	p = 0.8525
Apolipoprotein A-I (mg/mL)	Apo A-I	0.58-2.8	1.60 ± 0.29	1.63 ± 0.50	p = 0.9544	1.18 ± 0.79	2.10 ± 0.71	p = 0.1007
Apolipoprotein A-II (ng/mL)	Apo A-II	198-495	522.00 ± 48.67	397.00 ± 37.90	p = 0.1903	626.25 ± 169.56	528.00 ± 82.69	p = 0.1903
Apolipoprotein C-I (ng/mL)	Apo C-I	103-273	171.25 ± 24.45	169.25 ± 29.05	p = 0.9635	212.25 ± 87.39	279.75 ± 74.50	p = 0.2612
Apolipoprotein C-III (ug/mL)	Apo C-III	UN-185	85.33 ± 18.01	83.00 ± 0 .00	p = 0.4246	148.50 ± 63.75	203.25 ± 59.06	p = 0.2520
Brain-Derived Neurotrophic Factor (ng/mL)	BDNF	UN-3.5	0.595 ± 0.302	0.113 ± 0.056	p = 0.0262	0.70 ± 0.06	0.17 ± 0.10	p = 0.1703
C-Reactive Protein (ug/mL)	CRP	UN-0.95	0.051 ± 0.00	0.050 ± 0.007	p = 0.8056	0.285 ± 0.199	0.0370 ± 0.000	p = 0.0145
Complement C3 (mg/mL)	C3	0.33-1.2	0.365 ± 0.051	0.385 ± 0.031	p = 0.6387	0.703 ± 0.055	0.6250 ± 0.085	p = 0.1657
Factor VII (ng/mL)		8.5-165	25.5 ± 11.9	45.0 ± 16.1	p = 0.4775	154.50 ± 34.55	136.50 ± 27.40	p = 0.4775
Ferritin (ng/mL)	FRTN	UN-186	817.5 ± 645.7	960.0 ± 717.7	p = 0.9187	291.8 ± 199.1	153.0 ± 53.2	p = 0.9187
Fibrinogen (mg/mL)		0.061-.44	0.102 ± 0.020	0.131 ± 0.053	p = 0.4923	0.140 ± 0.016	0.125 ± 0.026	p = 0.5817
Growth Hormone (ng/mL)	GH	0.2-86	26.68 ± 21.60	15.28 ± 7.37	p = 0.4673	2.90 ± 2.97	12.33 ± 7.34	p = 0.4673
Haptoglobin (mg/mL)		UN-1.6	0.368 ± 0.271	0.560 ± 0.096	p = 0.6840	0.95 ± 0.509	0.743 ± 0.244	p = 0.6840
Insulin (μIU/mL)		UN-24	11.00 ± 2.55	3.93 ± 1.91	p = 0.0140	4.85 ± 0.55	4.53 ± 3.77	p = 0.7077
Interleukin-1 receptor antagonist (pg/mL)	IL-1ra	UN-199	1577.5 ± 273.6	1977.5 ± 366.8	p = 0.1308	343.0 ± 114.2	277.5 ± 124.8	p = 0.7479
Interleukin-6 (pg/mL)	IL-6	UN-19	6.03 ± 1.38	29.75 ± 16.24	p = 0.0081	4.40 ± 0.90	4.40 ± 0.00	p = 0.9185
Interleukin-8 (pg/mL)	CXCL8, IL-8	26-1160	272.75 ± 172.62	202.50 ± 72.24	p = 0.8709	184.25 ± 135.32	103.75 ± 19.51	p = 0.0181
Interleukin-12 Subunit p40 (ng/mL)	IL-12p40	UN-0.53	0.30 ± 0.00	0.22 ± 0.00	p = 0.5316	0.34 ± 0.10	0.28 ± 0.00	p = 0.5316
Interleukin-16 (pg/mL)	IL-16	UN-217	77.50 ± 3.64	82.00 ± 4.42	p = 0.7347	90.50 ± 27.80	52.25 ± 14.29	p = 0.0242
Interleukin-18 (pg/mL)	IL-18	UN-179	470.00 ± 165.83	915.25 ± 519.57	p = 0.1750	771.00 ± 122.09	715.00 ± 204.14	p = 0.8216
Macrophage-Derived Chemokine (pg/mL)	MDC	32-425	260.25 ± 94.23	174.25 ± 28.14	p = 0.3126	399.75 ± 153.23	250.75 ± 82.41	p = 0.1770
Macrophage Inflammatory Protein-1 beta (pg/mL)	MIP-1β, CCL4	178-2150	690.50 ± 120.88	1266.25 ± 1184.30	p = 0.6981	703.00 ± 109.86	1172.5 ± 1188.28	p = 0.6981
Matrix Metalloproteinase-2 (ng/mL)	MMP-2	2680-5100	4095.0 ± 778.38	4847.5 ± 1177.9	p = 0.5982	5090.0 ± 354.9	5285.0 ± 337.9	p = 0.8118
Matrix Metalloproteinase-3 (ng/mL)	MMP-3	0.8-3.2	0.820 ± 0.186	1.055 ± 0.102	p = 0.4452	1.675 ± 0.259	2.30 ± 0.648	p = 0.1119
Matrix Metalloproteinase-9 (ng/mL)	MMP-9	UN-349	46.33 ± 9.53	41.33 ± 3.77	p = 0.7166	68.50 ± 8.29	60.50 ± 14.84	p = 0.6900
Monocyte Chemotactic Protein 1 (pg/mL)	MCP-1, CCL2	UN-380	1084.25 ± 219.04	918.75 ± 276.21	p = 0.5085	341.75 ± 112.70	202.00 ± 49.56	p = 0.5085
Myeloperoxidase (ng/mL)	MPO	UN-1560	106.50 ± 32.23	98.50 ± 49.12	p = 0.8559	142.25 ± 58.98	115.5 ± 54.20	p = 0.7943
Myoglobin (ng/mL)		28-2400	442.75 ± 244.65	1211.0 ± 676.5	p = 0.0625	413.5 ± 253.3	269.5 ± 135.8	p = 0.6570
Neuron-Specific Enolase (ng/mL)	NSE	UN-1	0.557 ± 0.113	0.460 ± 0.228	p = 0.8814	0.568 ± 0.284	0.388 ± 0.084	p = 0.5622
Plasminogen Activator Inhibitor 1 (ng/mL)	PAI-1	3.2-128	49.50 ± 10.45	22.75 ± 10.06	p = 0.0084	16.58 ± 11.45	9.80 ± 2.01	p = 0.3895
Serotransferrin (mg/dL)	Transferrin	20-360	25.00 ± 2.00	33.00 ± 4.55	p = 0.9957	33.50 ± 3.28	34.00 ± 3.32	p = 0.9957
Serum Amyloid P-Component (ug/mL)	SAP	0.69-6.9	2.90 ± 0.35	3.40 ± 0.78	p = 0.4190	4.60 ± 0.99	3.03 ± 0.66	p = 0.0430
Stem Cell Factor (pg/mL)	SCF	UN-474	233.67 ± 26.40	300.50 ± 85.50	p = 0.9844	415.00 ± 150.77	322.75 ± 67.98	p = 0.5278
T-Cell-Specific Protein RANTES (ng/mL)	RANTES, CCL5	0.2-16	4.28 ± 1.73	0.90 ± 0.46	p = 0.0154	4.69 ± 4.40	1.38 ± 0.52	p = 0.0154
Thrombospondin-1 (ng/mL)		282-38600	5302.5 ± 2512.7	1089.3 ± 539.9	p = 0.0271	2743.0 ± 2450.8	1078.25 ± 409.45	p = 0.2763
Tissue Inhibitor of Metalloproteinases 1 (ng/mL)	TIMP-1	32-151	96.75 ± 8.84	83.50 ± 9.39	p = 0.1793	88.50 ± 15.11	66.25 ± 11.10	p = 0.0666
Transthyretin (mg/dL)	TTR	UN-13	ND	5.4 ± 0.00	ND	4.85 ± 0.75	7.05 ± 2.50	p = 0.1683
Tumor necrosis factor receptor 2 (ng/mL)	TNFR2	UN-4.9	7.53 ± 1.93	8.40 ± 1.88	p = 0.5647	11.90 ± 2.10	7.65 ± 1.19	p = 0.0277
Vascular Cell Adhesion Molecule-1 (ng/mL)	VCAM-1	209-389	555.75 ± 103.92	669.50 ± 67.45	p = 0.1521	576.50 ± 67.77	495.25 ± 34.07	p = 0.1958
Vascular Endothelial Growth Factor (pg/mL)	VEGF	UN-112	ND	60.00 ± 0.00	ND	60.00 ± 16.97	54.00 ± 0.00	p = 0.2136
Vitamin D-Binding Protein (ug/mL)	VDBP	UN-300	22.00 ± 4.00	24.25 ± 6.02	p = 0.2624	39.75 ± 4.09	45.75 ± 8.23	p = 0.2743
von Willebrand Factor (ug/mL)	vWF	87-429	360.25 ± 59.60	328.50 ± 89.30	p = 0.5729	205.25 ± 59.69	153.25 ± 42.48	p = 0.5729

Values in bold were significantly different between SIV + saline and SIV + deprenyl conditions. ND; not detected, UN; undetectable.

### Deprenyl decreases brain inflammatory gene expression

**Figure 5:**
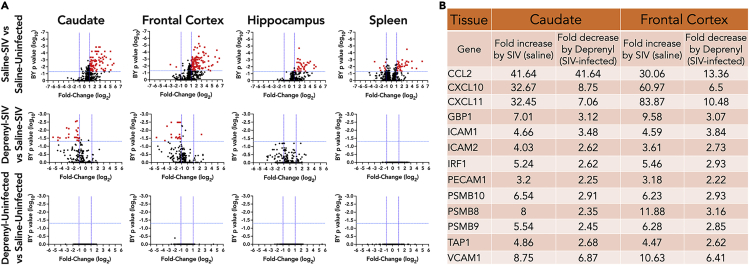
RNA was isolated from brain tissues and spleen from all SIV-infected animals (4 SIV + saline and 4 SIV + deprenyl) and analyzed for inflammatory gene expression using the nCounter Non-human Primate Inflammation panel (NanoString) Brain tissues analyzed included frontal gray matter, caudate, and hippocampus. SIV infection led to significant (>2-fold, corrected p value < 0.05) increases in numerous inflammatory genes in brain tissues and induced both up- and downregulated genes in spleen (A, upper row). Deprenyl treatment of SIV-infected animals significantly decreased many of these genes in caudate and frontal cortex, with no significant changes in hippocampus or spleen (A, middle row). Deprenyl had no effect on inflammatory gene expression in any tissues from uninfected animals (A, lower row). Examples of genes in the caudate and frontal cortex that are significantly upregulated by SIV and significantly downregulated in the presence of deprenyl are shown (B).

To assess the impact of deprenyl on the development of SIV-associated inflammation, RNA isolated from the frontal gray matter, caudate, hippocampus, and spleen at necropsy were analyzed for changes in inflammatory gene expression by using the NanoString nCounter Non-human Primate Inflammation panel (Table S2). In all three regions of the brain, SIV infection led to significant (>2-fold, corrected p-value <0.05) increase in numerous inflammatory genes (Figure 5A upper row). The spleen had both up- and downregulated genes (Figure 5A upper row). Treatment with deprenyl in SIV-infected animals led to significant changes in many of these genes in the caudate and frontal cortex, with no significant differences found in the hippocampus or spleen. Examples of genes significantly upregulated by SIV, and significantly downregulated in the presence of deprenyl, in the caudate and frontal cortex, are shown in Figure 5B. These are notable for chemokines (CCL2, CXCL10, and CXCL11), adhesion molecules (ICAM1, ICAM2, PECAM1, and VCAM1), interferon-inducible genes (GBP1, IRF1, and TAP1) including the immunoproteasome (PSMB8, PSMB9, and PSMB10), and antiviral genes (OAS2 and BST2). The full list of genes significantly (>2-fold, corrected p-value <0.05) altered by deprenyl in caudate and frontal cortex of SIV-infected animals is found in Table S3. None of these effects were seen in uninfected animals treated with deprenyl (Figure 5A, bottom row), suggesting that the anti-inflammatory activity of this drug may be specifically in response to SIV-associated neuroinflammation.

### Deprenyl and SIV alter monoamine metabolism

To verify that deprenyl was biologically active and functioning as expected under the conditions of this study, dopamine metabolism was assessed across different brain regions from all animals in the saline, deprenyl, SIV + saline, and SIV + deprenyl groups. These studies also enabled examination of alterations in regional brain biogenic amine and metabolite levels during SIV infection, something that has not been previously defined. Mood and anxiety disorders such as major depressive disorder and generalized anxiety disorder are highly comorbid with HIV (Beer et al., 2019; Camara et al., 2020; Nanni et al., 2015; Remien et al., 2019; Wang et al., 2018). As dysregulation of biogenic amine signaling has been implicated in the etiology of these disorders (Dunlop and Nemeroff, 2007; Lin et al., 2014; Mann, 2013; Mann and Currier, 2007), defining changes in biogenic amine levels may suggest mechanisms by which HIV infection could drive comorbid neuropsychiatric conditions.

As deprenyl is a monoamine oxidase inhibitor, it should prevent monoamine oxidase from metabolizing dopamine into its metabolite 3,4-dihydroxyphenylaceticacid (DOPAC). Sections of the caudate, putamen, substantia nigra, hippocampus, cerebellum, and frontal cortex were isolated from each animal, rapidly dissected in ice-cold HeGA to protect monoamines from oxidation, then processed and stored at −80°C until analysis by high-performance liquid chromatography (HPLC). Two samples were examined from each brain section in each animal, except for the substantia nigra, which was only large enough to generate a single sample from each animal. Samples were analyzed for concentration of the dopamine metabolite DOPAC, and for concentrations of dopamine and homovanillic acid (HVA), a downstream dopamine metabolite produced by catechol-*O*-methyltransferase mediated metabolism of DOPAC.

**Figure 6:**
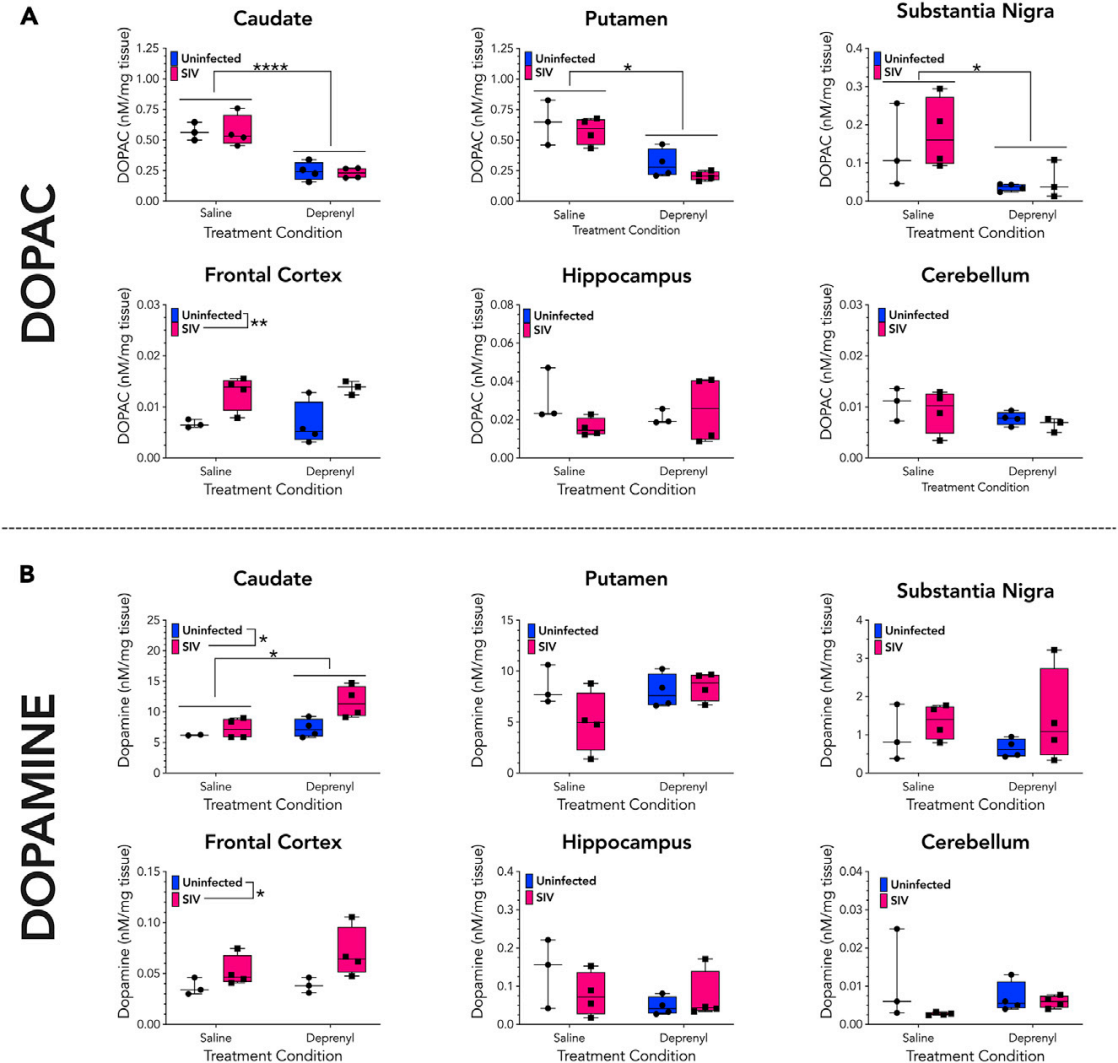
Brain sections were analyzed for monoamines and metabolites by HPLC (A). Two samples from each monkey were analyzed from the caudate, putamen, frontal cortex, hippocampus, and cerebellum of all SIV-infected animals (4 SIV + saline and 4 SIV + deprenyl) and averaged, while a single sample was examined from each substantia nigra. Each brain region was analyzed for the concentrations of dopamine metabolite 3,4-dihydroxyphenylacetic acid (DOPAC, A) and dopamine. (B). Treatment with deprenyl significantly decreased DOPAC concentrations in the caudate, putamen, and substantia nigra, while SIV, but not deprenyl, significantly increased DOPAC in the frontal cortex [A, 2-way ANOVA, SIV x deprenyl; (caudate; SIV, F (1, 11) = 0.0205, p = 0.889; deprenyl, F (1, 11) = 51.97, ∗∗∗∗p < 0.0001); (putamen; SIV, F (1, 11) = 1.979, p = 0.1871; deprenyl, F (1, 11) = 33.70, ∗∗∗p = 0.0001); (substantia nigra; SIV, F (1, 11) = 0.5178, p = 0.4883; deprenyl, F (1, 11) = 7.859, ∗p = 0.0187); (frontal cortex; SIV, F (1, 11) = 16.05, ∗∗ = 0.0025; deprenyl, F (1, 11) = 06713, p = 0.8008]. Assessment of dopamine concentrations showed that both SIV and deprenyl increased dopamine in the caudate, although there was no interaction. Additionally, SIV, but not deprenyl, significantly increased dopamine in the frontal cortex [B, 2-way ANOVA, SIV x deprenyl; (caudate; SIV, F (1, 10) = 6.711, ∗p = 0.0283; deprenyl, F (1, 10) = 6.711, ∗p = 0.0269); (frontal cortex; SIV, F (1, 10) = 5.360, ∗p = 0.0431; deprenyl, F (1, 10) = 2.168, p = 0.1717)].

In accordance with its activity as an MAO-I, deprenyl treatment significantly decreased DOPAC levels in the caudate, putamen, and substantia nigra (Figure 6A). This indicates deprenyl was biologically active, blocking the degradation of dopamine into DOPAC in dopamine-rich regions. In addition, we found that deprenyl treatment produced an increase in caudate dopamine levels but had no impact on dopamine levels in other brain regions (Figure 6B). Deprenyl treatment also did not affect levels of HVA, 5-HT, or 5-HIAA across any brain region tested (Figures S1, S2, and S3). As expected, dopamine concentrations were variable between brain regions, with the highest levels in striatal regions (caudate and putamen) and the lowest levels in the cerebellum. However, it was surprising to see that SIV infection increased frontal cortex DA, DOPAC, HVA, and 5-HT (Figures 6A, 6B, S1, and S2), increased caudate DA (Figure 6B), and increased substantia nigra 5-HT (Figure 6B). These results indicate that SIV infection alone results in gross disruptions to biogenic amine synthesis, signaling, and/or metabolism in the frontal cortex as well as less pronounced disruptions to caudate DA and substantia nigra 5-HT.

We did not observe any significant interaction between SIV infection and deprenyl treatment for dopamine, DOPAC, or HVA (Figures 6A, 6B, and S1). However, the SIV-induced increase in 5-HT levels in the frontal cortex was normalized by deprenyl treatment (Figure S2). In addition, deprenyl-induced elevations in putamen and frontal cortex 5-HIAA levels were absent in SIV-infected subjects (Figure S3). This suggests that deprenyl treatment similarly impacts dopamine indices in control and SIV-infected subjects, but may differentially impact serotonin synthesis, signaling, and/or metabolism depending on infection status.

## Discussion

Although efficacious antiretroviral therapy (ART) has transformed HIV infection into a chronic condition (Deeks et al., 2013a), PLWH can still develop HIV-related or other comorbid diseases. These comorbidities vary, but many are associated with chronic inflammation, which is prevalent in ART-treated PLWH (Deeks et al., 2013b; Negin et al., 2012; Pelchen-Matthews et al., 2018; Peterson and Baker, 2019; Yang et al., 2019). One frequent comorbidity is depression (Bhatia and Munjal, 2014; Hobkirk et al., 2015; Pinheiro et al., 2016; Wang et al., 2018), which is increasingly associated with peripheral inflammation (Miller and Raison, 2016). Therefore, therapeutics that simultaneously ameliorates inflammation and depression would be valuable adjuvants for ART. With this in mind, we examined the impact of the antidepressant deprenyl on SIV infection and SIV-associated inflammation during a reproducible infection phase, acute SIV infection. We found that deprenyl was remarkably potent in decreasing peripheral (in the blood plasma) and central (in the brain) inflammatory markers. No effect on viral load was found in either the periphery or CNS.

Deprenyl, also known as L-deprenyl or selegiline, was developed as an antidepressant in the 1960s, acting as a potent inhibitor of monoamine oxidase-B (MAO-B) (Knoll et al., 1965), the primary form of monoamine oxidase found in the human CNS. Deprenyl can also inhibit MAO-A in the CNS when delivered in formulations that bypass first-pass metabolism in the liver (Fowler et al., 2015), which would be the case in our experiment as it was injected, not given orally. Some data show monoamine oxidase inhibitors (MAOI) have pleiotropic effects that are not well defined (Kitani et al., 2001; Szoko et al., 2018) and which may be unrelated to monoamine oxidase inhibition (Koutsilieri et al., 1996; Kragten et al., 1998; Lieb, 1983; Nagatsu and Sawada, 2006; Ryu et al., 2018; Tabakman et al., 2004; Tatton et al., 1994). In animal models, these overlapping effects include regulation of inflammation and reactive oxygen species (ROS) (Bekesi et al., 2012; Bielecka et al., 2010; Morsali et al., 2013; Nagy et al., 2018; Tsao et al., 2014), alterations in gene expression (Tatton and Chalmers-Redman, 1996; Tatton et al., 1996), increasing longevity (Kitani et al., 1994, 1998; Knoll and Miklya, 2016), and promoting neurite outgrowth and neuronal survival (Abdanipour et al., 2018; Ebadi et al., 2002; Koutsilieri et al., 1994; Kragten et al., 1998; Morsali et al., 2013; Tazik et al., 2009). In the rodent heart, inhibiting monoamine oxidases reduces inflammation and endothelial dysfunction in the aorta (Ratiu et al., 2018; Sturza et al., 2013), decreasing ROS and preventing mitochondrial dysfunction and ER stress in diabetic cardiomyopathy (Deshwal et al., 2018). Also in rodents, inhibiting monoamine oxidase reduced LPS-mediated TNF-α and ROS production in a model of chronic wound formation (Ekuni et al., 2009). In rodent glia, the MAOI moclobemide reduced LPS-mediated increases in IL-1β, TNF-α, and NF-κB activity (Bielecka et al., 2010). The MAOI rasagiline decreased NLRP3 activation and IL-1β secretion in mouse bone-marrow-derived macrophages by inhibiting production of mitochondrial ROS and subsequent NF-κB activation (Sanchez-Rodriguez et al., 2020).

There are relatively few studies examining MAOI in humans or human cell models of inflammatory disease. Moclobemide reduces LPS-induced increases in TNF-α and IL-8 in human blood (Lin et al., 2000) and deprenyl blocks increases in IL-8, ROS production, and NF-κB activation in human airway epithelial cells exposed to cigarette smoke medium (Cui et al., 2017). In a series of case reports, MAOI were shown to reduce the symptoms of rheumatoid arthritis (Lieb, 1983), and deprenyl acts through an MAO-independent mechanism to reduce mitochondrial respiration and ROS production in human myeloid leukemia cells (Ryu et al., 2018). In the context of HIV, the impact of treatment with transdermal selegiline (*i.e.* deprenyl) on cognitive function was examined in a clinical trial (ACTG5090), although this trial did not examine the impact on inflammation. In this trial, a primarily male, majority-white cohort of 128 HIV-infected, ART-treated individuals with cognitive impairment showed no cognitive improvement after 24 weeks of transdermal deprenyl treatment, although modest improvements in several cognitive domains were found at 24 and 48 weeks (Evans et al., 2007; Schifitto et al., 2007). Analyses of a subgroup using proton magnetic resonance spectroscopy showed deprenyl did not decrease oxidative stress or ameliorate brain metabolite changes, nor abnormalities associated with HIV infection (Schifitto et al., 2009). Notably, the trial showed no effect of deprenyl on viral load after 24–48 weeks, and inflammatory biomarkers were not examined in this trial, so the impact of deprenyl on inflammation is not clear. Our findings corroborate a lack of effect on viral replication, showing that deprenyl had no effect on SIV load in the plasma, CSF, brain, or spleen. However, deprenyl did show broad anti-inflammatory effects in the CNS and periphery at 1–2 weeks post infection. Notably, our study shows that deprenyl did not suppress inflammatory biomarkers or gene expression in mock-infected macaques, suggesting the anti-inflammatory impact may be specifically in response to the inflammation induced by SIV infection.

In the periphery, deprenyl treatment lowered the plasma level of many inflammatory biomarkers that were increased in response to SIV infection. For example, CRP was increased significantly in the saline-treated SIV-infected animals at day 14 but was not elevated in the infected animals administered deprenyl. Similarly, the SIV-induced elevation of TNFR2 was ablated by deprenyl. Effects on immune mediators were also present, as exemplified by the chemokines IL-8 and CCL5. Deprenyl also affected peripheral immune cells, with reductions in the CD8^+^ T cell and inflammatory monocyte populations.

In the CNS, deprenyl mediated substantial reductions in SIV-infected inflammatory gene expression in the frontal gray matter and caudate. This is exemplified in the changes in the gene expression of the chemokines CCL2, CXCL10, CXCL11, and CCL1 in the brain. For CCL2, which drives monocyte transmigration into the CNS and is important to the development of neuroHIV, SIV infection increased expression in both the caudate and frontal cortex, and deprenyl treatment decreased CCL2 expression in both regions in the infected animals. Similar findings occurred for the T cell chemoattractants CXCL10 and CXCL11. Interestingly, while SIV infection significantly increased CXCL10 and CXCL11 in the hippocampus, it did not increase CCL2 in this area, suggesting regionally specific responses to infection.

Further, deprenyl did not significantly decrease CXCL10 or CXCL11 (or other increased genes) in the hippocampus as it does in the frontal cortex or caudate, also indicating regional-specific responses in the brain to deprenyl. In contrast to CCL2, CXCL10, and CXCL11, CCL1, a chemoattractant for monocytes and T lymphocytes, was one of the two genes that was significantly downregulated in the frontal cortex of SIV-infected animals. Interestingly, deprenyl significantly upregulated CCL1 expression in that brain region in infected animals. These data suggest that the anti-inflammatory effects of deprenyl are not inherent to this drug but are based on the environment and only occur in response to specific inflammatory changes. This also suggests that the anti-inflammatory impact of MAOIs may be more challenging to evaluate, as the effects are situational.

The mechanism(s) by which deprenyl suppresses inflammation, particularly peripheral inflammation, is not well understood. As noted above, deprenyl and other MAO-I have broad anti-inflammatory effects in both rodent and human systems. These effects may be unrelated to their impact on monoamine oxidases (MAOs), but they could also be associated with inhibition of MAO activity in various cell types. The peripheral bioactivity of deprenyl is not defined, but both types of MAO are expressed in the periphery, across many organ systems and in several types of immune cells, although the level of expression and activity is considerably less than in the CNS (Billett, 2004; Rodriguez et al., 2001; Sivasubramaniam et al., 2003). The effects of deprenyl on inflammation may also vary between the CNS and periphery, and among peripheral organs, depending on the cell composition of the compartment being investigated, as the effects on populations with high levels of T and B cells are likely different than the effects on populations of macrophages, neurons, and glia.

Specifically in the context of SIV, studies by Czub et al. have examined the impact of selegiline (*i.e.* deprenyl) on SIV-infected macaques. These data show that deprenyl treatment begun at two weeks after virus inoculation, at the peak of acute viremia, increased the extent of neuropathology, the number of SIV-infected cells, and inflammatory cytokine production in the brain. These changes were primarily observed in the basal ganglia, frontal cortex, and hippocampus, and included increased vacuolization, focal accumulations of lymphocytes and microglia cells, and an increase in mRNA for TNF-α (Czub et al., 2001, 2004). These effects were specifically linked to increases in CNS dopamine, caused by a decrease in the activity of both MAO-A and MAO-B activity, consistent with the administration of the drug by injection, as discussed above. Our prior data also show that dopamine increases HIV infection and inflammation in human macrophages and microglia, central drivers of HIV neuropathogenesis (Nickoloff-Bybel et al., 2019; Nolan et al., 2020). We have also shown that methamphetamine (Meth), which increases dopamine, exacerbates various aspects of CNS disease resulting from SIV infection in non-human primates (Marcondes et al., 2010; Najera et al., 2016; Niu et al., 2020), suggesting that dysregulated dopamine has a deleterious effect on neuroHIV (Nickoloff-Bybel et al., 2020).

In these experiments, deprenyl treatment increased CNS tissue levels of dopamine in the caudate and decreased DOPAC levels in the caudate, putamen, and substantia nigra, demonstrating that our deprenyl treatment functioned as expected. Interestingly, while we did not observe any significant interaction between deprenyl treatment and cortical dopamine indices, deprenyl did normalize prefrontal cortex serotonin tissue levels that were elevated following SIV infection. Serotonin can influence cognitive function (Schmitt et al., 2006), but has a more well defined role in effect and mood disorders (Lin et al., 2014; Mann, 2013). This suggests that deprenyl may be more effective in the treatment of mood disorders than cognitive deficits associated with HIV infection. The aforementioned clinical trial (ACT5090) did not report any investigation of affect or mood disorders (Evans et al., 2007; Schifitto et al., 2007), suggesting a need for studies that characterize the efficacy of deprenyl treatment on affect or mood disorders that are often comorbid with HIV infection (Beer et al., 2019; Camara et al., 2020; Nanni et al., 2015; Remien et al., 2019; Wang et al., 2018).

Surprisingly, we also found that SIV infection itself increased DA, DOPAC, HVA, and 5-HT in the frontal cortex, increased dopamine in the caudate, and increased 5-HT in the substantia nigra. Elevations in dopamine and 5-HT levels may be a product of increased synthesis of these neurotransmitters and increases in synthesis may subsequently result in greater metabolism and an elevation in metabolite levels. Studies in SIV-infected rhesus macaques and HIV-infected humans have also shown increases (Koutsilieri et al., 1997; Scheller et al., 2010) in dopamine and metabolite levels in the CSF. Thus, alterations in biogenic amine and metabolite levels observed in SIV infected monkeys may be a product of increased synthesis. However, this hypothesis is inconsistent with others studies showing decreased dopamine metabolite levels in the brain (Kumar et al., 2009) and CSF (di Rocco et al., 2000; Larsson et al., 1991; Saloner et al., 2020) of people living with HIV, as well as human PET imaging studies showing reduced DA signaling indices that correlate with cognitive symptoms in patients with HIV (Chang et al., 2008).

Alternatively, SIV-induced reductions in biogenic amine release may alter the compartmental distribution of biogenic amines and metabolites, resulting in increased tissue stores of these compounds. Brain biogenic amines and their metabolites are found in three separate compartments: (1) the cytosol of terminals, (2) vesicles within the cytosol of terminals, and (3) the extracellular space. Decreased biogenic amine release into the extracellular space due to reduced neuron firing, autoreceptor inhibition, or a disruption in vesicular packaging or release mechanisms could shift in the distribution of biogenic amines from the extracellular pool to either the cytosolic or vesicular pools (Best et al., 2009, 2010). Postmortem brain tissue collection procedures isolate the cytosolic and vesicular pools from the extracellular pool, thereby measuring only the biogenic amines and metabolites contained in the cytosolic and vesicular compartments. Thus, the observed reductions in dopamine and serotonin release would correspond with increases in brain tissue levels. This mechanism is consistent with preclinical studies showing that the HIV Tat protein can disrupt biogenic amine vesiculation by inhibition of the vesicular monoamine transporter (Midde et al., 2012), and that transgenic rats expressing HIV proteins display reduced dopamine and serotonin release capacity (Denton et al., 2019). Future studies should examine the mechanism by which SIV infection disrupts biogenic amine signaling, but these data indicate that SIV infection alone can disrupt biogenic amine processing and that the frontal cortex is particularly susceptible to this effect.

In contrast to prior macaque studies, the increases in dopamine we observed did not increase CNS viral load in the caudate or frontal cortex. Further, we observed decreased inflammatory gene expression in this region relative to increased inflammation in response to deprenyl and other dopamine-elevating drugs such as L-DOPA (Czub et al., 2001, 2004). Increases in neuropathology and CNS viral loads were also seen in macaques in response to Meth (Marcondes et al., 2010; Najera et al., 2016; Niu et al., 2020). The reason for the differences is not clear, but the distinct pathological effects of Meth, and other addictive substances, may be due to the substantially greater magnitude of dopamine release. In this study, in SIV-infected macaques, deprenyl increased caudate and frontal cortex dopamine by 59% and 56%, respectively. In contrast, amphetamine treatment in rhesus macaques increased dopamine 1000%–6000% in caudate and 600%–2000% in PFC as measured by microdialysis (Jedema et al., 2014), while a separate study showed direct injection of d-amphetamine into the caudate and PFC increased dopamine in each region by 1000%–1500% or 200%–500%, respectively (Saunders et al., 1994). The large difference between Meth and deprenyl-evoked dopamine release suggests that the differential pathology seen in these studies is due to the different magnitude of change in dopamine release. Also, direct comparison of these studies requires some caution, due to differences in experimental methodology, no specific report of dopamine concentrations in Meth studies and additional effects of Meth, such as impacts on blood–brain barrier permeability (Turowski and Kenny, 2015) and/or activation TAAR-1 receptors (Underhill et al., 2021).

The distinct timing and length of deprenyl treatment may also contribute to the differences between these studies and prior studies of SIV neuropathogenesis in deprenyl-treated macaques. In the current study, deprenyl treatment started one week prior to SIV inoculation and was maintained until 2 weeks post infection to examine the impact of deprenyl on the development of acute infection. The previous studies began treatment with deprenyl at two weeks post SIV inoculation and assessed output parameters at 8–20 weeks post infection. As the effects of deprenyl may be situational, the MAOI and non-MAOI-related effects of this drug could have been triggered by different environmental conditions, inflammatory milieu, and/or stage of disease at the time of treatment. Further studies delineating the specific mechanisms by which deprenyl can act will be needed to resolve these questions.

Another critical consideration is that the anti-inflammatory effects of deprenyl, while seemingly beneficial, were only seen during the acute phase of infection. Importantly, these acute anti-inflammatory effects could be detrimental to long-term disease outcomes. Some of the genes that were downregulated by deprenyl in the CNS were antiviral genes, including OAS2 and tetherin (BST2). Several other antiviral genes (MX1, MX2, and OAS1) were also significantly upregulated by SIV and downregulated more than 2-fold by deprenyl, although the deprenyl mediated decrease in these latter genes did not reach significance. Decreases in CNS antiviral genes could certainly result in the development of a more robust infection in this compartment, and a recent study noted an inverse correlation in expression of MX1, OAS1, and MX2 expression with SIV RNA levels in the CNS (Mohammadzadeh et al., 2021).

Previous studies in macaques have also shown that several of our results, including reduction in CD8^+^ T cells (Marcondes et al., 2015), decreases in IL-16 (Amiel et al., 1999) and an early blockade of inflammatory factors such as interferon stimulated genes (Sandler et al., 2014) can worsen disease progression in macaques. Notably, although CD8^+^ T cells are decreased by deprenyl, they are not reduced to the level induced in CD8-depletion experiments, so it is not clear that deprenyl-mediated CD8 reductions would result in the same sort of pathology. We also found a deprenyl-mediated increase in IL-6 at day 7, but this returned to baseline levels by day 14. We previously reported that IL-6 is increased in the periphery and CNS during SIV infection (Roberts et al., 2004), and that this is altered by depletion of CD8 cells (Madden et al., 2004). Other groups have also shown increased IL-6 correlates with more severe CNS disease in macaques (Gopalakrishnan et al., 2021; Witwer et al., 2009). However, the effects of increased IL-6 are mostly seen in respect to longer term elevation of this cytokine. Thus, the transient nature of the increase we observed in IL-6 makes it unclear whether this would have any impact on disease progression. Longitudinal studies examining disease course and changes in inflammatory factors over time will be required to determine if the impact of deprenyl-mediated anti-inflammatory effects is beneficial or deleterious in the longer term.

### Limitations of the study

In addition to the potential confounds associated with the timing of deprenyl treatment and the potential for decreases in inflammation to worsen disease progression, this study has several other important limitations. As with many non-human primate studies, the sample size (n = 4) is a significant caveat, particularly because disease course and associated inflammation in both PLWH and SIV-infected macaques is robustly heterogeneous, with widely varying inflammatory effects and viral loads in untreated animals at the same timepoint during chronic disease. This small number of animals used in this study makes the results more susceptible to inter-animal heterogeneity and single outliers, particularly when correcting for multiple analyses or when the effect sizes are moderate or variable. To ameliorate somewhat these issues, macaques were sacrificed at two weeks post infection. In SIV infection, two weeks post infection is the most reproducible time in terms of viremia, as it generally represents peak viremia. This is predictive of set-point viremia and has much lower variability than measuring set-point and later levels of virus (Parker et al., 2001).

A second caveat is that the gene expression changes were defined using an immunology expression panel, so it is possible that other “non-immune”-associated genes were missed in this analysis. Many types of such genes, such as those regulating cellular metabolism, also strongly influence inflammatory activity, so a wider analysis of deprenyl-induced changes to gene expression in the context of SIV could improve our understanding of the pathways through which deprenyl downregulated inflammatory activity. Furthermore, genes and pathways involved in neuronal function were not studied and we only examined three brain regions and the spleen, while other areas of the brain and peripheral organs that can affect the brain and disease course were not examined.

## Summary

This study shows that the monoamine oxidase inhibitor deprenyl, an antidepressant with a long history of use in depression and Parkinson disease, has broad anti-inflammatory effects when given during the acute stage of SIV infection. Inflammation is a central factor in HIV-associated morbidity in the current era (Deeks et al., 2013b) and CNS inflammation is common in early infection and associated with the development of neuroHIV (Saylor et al., 2016; Valcour et al., 2012). These data suggest deprenyl as an attractive candidate for drug repurposing, as there is a critical need for new anti-inflammatory ART-adjuvants. Furthermore, the prevalence of depression in PLWH is high, deprenyl has a strong safety profile in HIV-infected individuals on ART (Evans et al., 2007; Schifitto et al., 2007), and its capacity to be administered transdermally improves longitudinal adherence and increases tolerability (Asnis and Henderson, 2014). These data also indicate that the anti-inflammatory mechanisms of MAOI class drugs deserve more attention, particularly with the growing amount of data linking inflammation to depression and the potential importance of anti-inflammatory treatments (Beurel et al., 2020; Dantzer et al., 2008; Kohler et al., 2016). A better understanding of these mechanisms could enable use of these well-known drugs for a variety of diseases, including HIV, and provide important insight into previously undefined pathways mediating inflammation.

## STAR★Methods

### Key resources table

**Table:** 

REAGENT or RESOURCE	SOURCE	IDENTIFIER
**Antibodies**		

BV605 Anti-mouse/human CD11b, clone M1/70	BioLegend	Cat# 101257; RRID: AB_2565431
BV421 Mouse anti-human CD3, clone SP34	BD Biosciences	Cat# 562877; RRID AB_2737860
BV786 Mouse anti-human CD4, clone L200	BD Biosciences	Cat# 563914; RRID AB_2738485
PeCy7 Mouse anti-human CD8, clone RPAT8	BD Biosciences	Cat# 557746; AB_396852
ECD Mouse anti-human CD14, clone RM052	Beckman Coulter	Cat# IM2707U; RRID AB_130853
APCH7 Mouse anti-human CD16, clone 3G8	BD Biosciences	Cat# 560195; RRID AB_1645466
BV711 Mouse anti-human CD20, clone 2H7	BD Biosciences	Cat# 563126; RRID AB_2313579
Iba-1, rabbit polyclonal antibody	Fujifilm Wako Pure Chemical Corporation	Cat# 019-19741; RRID AB_839504
ImmPress – HRP Horse Anti-Rabbit IgG Polymer Reagent	Vector Laboratories	Cat# MP-7401; RRID AB_2336529

**Bacterial and virus strains**		

SIVmac251 (passaged through brain microglia and Chinese origin rhesus macaques)	H.S Fox, UNMC, Omaha, NE	N/A

**Chemicals, peptides, and recombinant proteins**		

Glacial Acetic Acid, HPLC grade	Thermo Fisher	A35-500
TRIzol	Life Technologies	Cat# 15596026
DNTPs	Invitrogen	Cat# 18091050
SuperaseIN	Invitrogen	Cat# AM2696
Superscript/5X Buffer/0.1M DTT: Kit	Invitrogen	Cat# 18090010
Red blood cell lysis buffer	Sigma Aldrich	Cat# 11814389001
Nova Red	Vector Laboratories	Cat# SK-4805
Hematoxylin QS	Vector Laboratories	Cat# H-3404
Flow cytometry Buffer	eBioscience	Cat# 00-422-26
Brilliant stain buffer	BD Biosciences	Cat# 563794

**Critical commercial assays**		

nCounter NHP Immunology expression panel	NanoString	Cat#NS_NHP_IMMUNOLOGY_V2.0
Taqman RNA-to-CT One-Step Kit - RNA (qPCR)	Thermo Fisher Scientific	Cat# 4392938
one-step RT ddPCR Advanced kit for probes (500 rxns)	BioRad	Cat# 1864022
Qiagen RNeasy Mini Kit	Qiagen	Cat# 74106
QIAamp Viral RNA mini kit	QIAGEN	Cat# 52906
Live Dead assay UV blue	Invitrogen	Cat# L23105
2.5 HD RED Assay	Advanced Cell Diagnostics	Cat# 322360

**Experimental models: Organisms/strains**		

Chinese origin rhesus macaques	PrimGen	http://www.primgen.com/

**Oligonucleotides**		

Primer probe set MX1	Thermo Fisher Scientific	Cat# Rh02842279_m1
Primer probe set ALAS1	Thermo Fisher Scientific	Cat# Rh02829381_m1
Primer probe set CXCL11	Thermo Fisher Scientific	Cat# Rh02621763_m1
Primer probe set OAZ1	Thermo Fisher Scientific	Cat# Rh02809570_gH
Primer probe set IRF1	Thermo Fisher Scientific	Cat# #Rh00971962_m1
Primer probe set OAS2	Thermo Fisher Scientific	Cat# Rh02842591_m1
SIV qRT-PCR forward primer (5’-GTCTGCGT CATCTGGTGCATTC-3′)	Integrated DNA Technologies	Custom
SIV qRT-PCR reverse primer (5’-CACTAGGTG TCTCTGCACTATCTGTTTTG-3′)	Integrated DNA Technologies	Custom
SIV qRT-PCR probe (5’-/56-FAM/CTT CCT CAG/ZEN/TGT GTT TCA CTT TCT CTT CTG CG/3IABkFQ/- 3′)	Integrated DNA Technologies	Custom
TBP Forward: aaagaccattgcacttcgtg	Eurogentec	Custom
TBP Reverse: ggttcgctctcttatcc	Eurogentec	Custom
TBP Probe: tcccaagcggtttgctgcag	Eurogentec	Custom
RNAscope SIV probes	Advanced Cell Diagnostics	Cat# 317221

**Software and algorithms**		

BD FACSDiva v9.0	BD Biosciences	https://www.bdbiosciences.com/en-eu/products/software/instrument-software/bd-facsdiva-software#Overview
FlowJo for Mac version 10.6	BD Biosciences	https://www.flowjo.com/citing-flowjo
Graph Pad Prism 9.0 software	Graph Pad	https://www.graphpad.com/scientific-software/prism/

### Resource availability

#### Lead contact

Further information and requests for resources and reagents should be directed and will be fulfilled by the lead contact, Peter J. Gaskill (pjg63@drexel.edu).

#### Material availability

This study did not generate new unique reagents.

### Experimental model and subject details

#### Rhesus macaques, deprenyl treatment and SIV infection

Sixteen male rhesus macaques (*Macaca mulatta*) of Chinese origin, which were SIV, simian retrovirus type D, simian T cell leukemia virus, and herpes B (*Macacine alphaherpesvirus 1*) virus-free were purchased from PrimGen (Hines, IL). The macaques were an average of 5 years old at necropsy, with a range of 3.5–6.75 years of age. All experiments had the approval from University of Nebraska Medical Center and Drexel University Institute Animal Care and Use Committees and followed the animal use guidelines set by the National Institutes of Health. Macaques were housed in compliance with the Animal Welfare Act and the Guide for the Care and Use of Laboratory Animals in the non-human primate facilities at Department of Comparative Medicine, University of Nebraska Medical Center (UNMC). The primate facility at UNMC has been accredited by the AAALAC international. This study was reviewed and approved by the UNMC Institutional Animal Care and Use Committee under protocol 16-020-04-FC entitled “Mechanism of drug abuse mediated SIV neuropathogenesis and immune dysregulation” approved 4/16/2016. Animals were maintained in a temperature-controlled (23 ± 2°C) indoor climate with 12-h light/dark cycle and fed Teklad Global 25% protein primate diet (Envigo, St. Louis, MO) supplemented with fresh fruit or vegetables, and water *ad libitum*. The monkeys were observed twice daily for health status by animal care and veterinary personnel.

Following quarantine, animals were acclimated to intramuscular injections for 2–3 weeks using positive reinforcement. Animals, kept in containment, were anesthetized with 10–15 mg/kg of ketamine intramuscularly before procedures. Animals were treated with vehicle (saline) or 2 mg/kg R-(−)-Deprenyl hydrochloride in saline (Sigma-Aldrich, St. Louis, MO) starting 1 week prior to infection as described in the Results. Animals were inoculated with 10^7^ RNA copies of a stock SIVmac251 prepared in laboratory of Howard Fox, derived by serial passage *in vivo* through brain microglia and then through the blood of Chinese origin macaques (Burdo et al., 2005). The stock of SIVmac251 was diluted in a total of 3 mL RPMI 1640 (Invitrogen, Carlsbad, CA) and animals inoculated intravenously. Necropsy was performed 14 days after SIV inoculation by terminal anesthesia using overdose of ketamine and xylazine, consistent with the recommendations of the Panel on Euthanasia of the American Veterinary Medical Association. To protect monoamines in the brain tissue from oxidation, at necropsy, macaques were intracardially perfused with ice-cold 50% HeGA (glacial acetic acid (10^−1^ M), EDTA (10^−4^ M), 0.12% glutathione)/50% isotonic saline solution (pH 6). Brains were removed and rapidly dissected on ice, separating caudate, putamen, substantia nigra, corpus callosum, cerebellum, hippocampus, and frontal cortex.

### Method details

#### SIV plasma viral load quantification

SIV RNA in the plasma was quantified by using quantitative reverse transcription-PCR (qRT-PCR) using our established protocols (Acharya et al., 2020). Plasma was separated from blood samples collected in K2-EDTA vacutainer tubes (Becton Dickinson, San Diego, CA) within 4 h of collection. CSF was collected by cisterna magna puncture using a 22-gauge needle and dripped into a microfuge tube. RNA was extracted from 140 μL of plasma or CSF using a QIAamp Viral RNA mini kit according to manufacturer’s instructions (QIAGEN Germantown, MD). RNA was eluted in 50 μL of Buffer AVE. SIV gag RNA was quantified by qRT-PCR using the TaqMan RNA-to-Ct 1-Step Kit (Thermo Fisher Scientific, MA and Applied Biosystems QuantStudio3 Real-Time PCR System (Applied Biosystems, Waltham, MA). The reaction mix contained 12.5 μL of 2X Taq Buffer, 0.63 μL of 40X enzyme, 7.12 μL of nuclease-free water (Invitrogen, San Diego CA), 1 μL of 10 μM forward primer (5′-GTCTGCGTCATCTGGTGCATTC-3′), 1 μL of 10 μM reverse primer (5′-CACTAGGTGTCTCTGCACTATCTGTTTTG-3′), and 0.25 μL of 10 μM probe (5’–/56-FAM/CTT CCT CAG/ZEN/TGT GTT TCA CTT TCT CTT CTG CG/3IABkFQ/–3′), all custom from Integrated DNA Technologies (IDT), Coralville, IA. The thermal cycling conditions of the reaction were 48°C for 15 min, 95°C for 15 min and 40 cycles of 95°C for 15 s and 60°C for 1 min (data collection) (Byrareddy et al., 2014; Cline et al., 2005).

#### Flow cytometry

Flow cytometry evaluation was completed on peripheral whole blood from all study subjects. Blood was assessed at 2 weeks prior and the equivalent of day 0, 7 and 14 post SIV infection. All cells and staining reagents were kept on ice throughout staining procedure. Centrifugation steps were conducted at 4°C – 3500 × g. Whole blood (200 μL) was washed in Dulbecco’s phosphate buffered saline (dPBS) and stained for 30 min with UV blue Live dead assay (Invitrogen, Carlsbad, CA). Post live dead stain, whole blood was washed and then blocked using flow cytometry buffer (eBioscience, Waltham, MA). Cells were pelleted and surface epitopes were stained for 45 min with a cocktail containing brilliant stain buffer and fluorochrome-conjugated monoclonal antibodies against CD3-BV421, CD4-BV786, CD8-PeCy7, CD11b-BV605, CD14-ECD, CD16-APCH7, and CD20-BV711. After surface epitope staining, cells were washed with flow cytometry buffer, pelleted and red blood cells were lysed a total of three times with red blood cell lysis buffer (Sigma Aldrich, St. Louis, MO). Lysed whole blood was washed in flow cytometry buffer, pelleted, and fixed using 1% paraformaldehyde in flow cytometry buffer. Cells were held overnight at 4°C before acquisition. All events were acquired using the BD Fortessa X450, and data were analyzed using BD FACSDiva v9.0 or FlowJo for Mac version 10.6 (BD Biosciences, Ashland, OR).

Complete blood counts were performed in the CLIA-certified Pathology labs of Nebraska Medicine. The lymphocyte percentage was determined as proportions of white blood cells. The lymphocyte percentage was multiplied by the white blood cell count to obtain lymphocytes per microliter, and further multiplied by the flow cytometric determination of the CD3^+^CD4^+^ and CD3^+^CD8^+^ proportions within the lymphocyte FSC/SSC gate on live cells to obtain the number of CD4^+^ and CD8^+^ T-cells, respectively, per microliter of blood. For the monocytes, the proportion of CD14 + CD16 ^+^ cells within the FSC/SCC gate on live cells of the total live cells within this gate was reported.

#### Analysis of peripheral biomarkers

For analysis of analytes in peripheral blood, EDTA-anticoagulated plasma was collected at necropsy and sent to Myriad RBM (Austin, TX) for analysis using the Human v2.0 Multianalyte profile (MAP) immunoassay. The assay examined 85 total analytes, which are listed in Table S1. The 46 analytes that were detected in our macaques and the normal ranges determined by analysis of plasma from 39 healthy Rhesus macaques, are shown in Table 1. Although these MAPs are designed for human samples, Myriad RBM assays have been previously used for successful analysis of non-human primate inflammation and neurological disease (Byrnes-Blake et al., 2012; Dietsch et al., 2015; Gautam et al., 2018; Kobayashi et al., 2013; Olsen et al., 2010; Shen et al., 2016). However, for analytes with all undetectable readings, these may either be due to lack of cross-reactivity or the absence of those molecules in the samples tested. All samples were stored at less than −75°C until tested. Samples were thawed at room temperature, vortexed, spun at 3700 × g for 5 min for clarification and transferred to a master microtiter plate. Using automated pipetting, an aliquot of each sample was added to individual microsphere multiplexes of the Human v2.0 MAP and blocker. This mixture was thoroughly mixed and incubated at room temperature for 1 h. Multiplexed cocktails of biotinylated reporter antibodies were added robotically and after thorough mixing, incubated for an additional hour at room temperature. Multiplexes were labeled using an excess of streptavidin-phycoerythrin solution, thoroughly mixed and incubated for 1 h at room temperature. The volume of each multiplexed reaction was reduced by vacuum filtration and washed 3 times. After the final wash, the volume was increased by addition of buffer for analysis using a Luminex instrument and the resulting data interpreted using proprietary software developed by Myriad RBM. For each multiplex, both calibrators and controls were included on each microtiter plate. Eight-point calibrators to form a standard curve were run in the first and last column of each plate and controls at three concentration levels were run in duplicate. Standard curve, control, and sample QC were performed to ensure proper assay performance. Study sample values for each of the analytes were determined using weighted and non-weighted curve fitting algorithms and 4 and 5 parameter logistics.

#### Analysis of monoamines and metabolites

Isolation of brain tissue was performed carefully in anoxic conditions to prevent oxidation of dopamine and enable its analysis by HPLC as described above. Tissue (∼200 mg) from brain sections collected on ice was placed in ice-cold, pre-filtered HeGA. Samples were disrupted and homogenized using an ultrasonic homogenizer (Model 300VT, Biologics, Inc., Manassas, VA) at 80% power, with 2 s pulses over 15 s followed by 5 s off and repeated a total of six times. Homogenized tissues were aliquoted and stored at −80°C. For HPLC analysis, samples were thawed on wet ice, and then centrifuged for 4 min at 16,000 × g at 4°C. The supernatant was removed and stored at 4°C until sampled for analysis using HPLC coupled to electrochemical detection (no more than 12 h). From 3 to 10 μL samples were drawn once every 35 min using an autosampler (Shimadzu, Kyoto, Japan). Separation was achieved as previously described (Brodnik et al., 2017a, 2017b; Brodnik and Jaskiw, 2015), using a 100 × 3-mm reversed-phase C18 column with 3-μm particles (Phenominex Luna C-18 (2), CA, USA) column. The mobile phase consisted of 12.5 mM citrate, 20 mM acetate, and 0.1 mM EDTA, with 5% (v/v) methanol adjusted to pH 6.0 with sodium hydroxide and 0–3.0 mM octyl sulfonic acid adjusted as a modifier. Dopamine (DA), serotonin (5-HT), and the metabolites 3,4-Dihydroxyphenylacetic acid (DOPAC), 5-Hydroxyindoleacetic acid (5-HIAA) and homovanillic acid (HVA) were quantified with a glassy carbon electrode maintained at a relative potential of 0.65 V to an Ag/AgCl reference electrode (Antec Decade Elite, Zoeterwoude, The Netherlands).

#### SIV cell-associated RNA quantification from frozen tissues

Total cell associated SIV RNA was quantified using RT-ddPCR using 100 ng of RNA, One-Step RT-ddPCR Advanced Kit for Probes (Bio-Rad, Hercules, CA) and same set of primers and probe used for SIV plasma viral load quantification; ddPCR was carried out on Bio-Rad QX200 AutoDG digital droplet PCR system as described previously (Cline et al., 2005). In brief, 22 μL of reaction mix was used for droplet generation using QX200 Droplet Generator. The 22 μL of reaction mix contains 5 μL of super mix, 2 μL of reverse transcriptase, 0.5 μL of 20 μM forward primer, 0.5 μL of 20 μM reverse primer, 0.25 μL of 20 μM probe, 1.0 μL of 300 mM DDT, 2.5 μL of Nuclease Free Water and the rest template RNA. The ddPCR plate with the emulsified samples was heat sealed and amplified in a C1000 Touch thermal cycler (Bio-Rad, Hercules, CA). The thermal cycling condition of the reaction was 48°C for 1 h, 95°C for 10 min, 45 cycles of 94°C for 30 s and 59°C for 1 min, 98°C for 10 min and hold at 12°C. After thermal cycling, ddPCR plates were transferred to the QX200 droplet reader (Bio-Rad, Hercules, CA) for droplet count and fluorescence measurement. Positive droplets with amplified products were separated from negative droplets without target amplicon by applying a fluorescence amplitude threshold and the absolute quantity of RNA per sample (copies/μL) was determined using QuantaSoft software (Bio-Rad, Hercules, CA).

#### Immunohistochemistry and RNAscope

Tissues were fixed in 10% neutral buffered formalin for 48 h, transferred to 70% ethanol, and processed for paraffin embedding. Five μm thick tissue slices were dehydrated at 60°C, deparaffinized and rehydrated in a series of xylene and graded ethanol (100%, 95%, 75%, 50%, dPBS). Slides were rinsed with dPBS and blocked for endogenous peroxidases using 3% H_2_O_2_ in methanol, slides were again rinsed in dPBS. Heat induced antigen retrieval was completed by submerging sections in 0.1 M sodium citrate, pH 6.39 and heated in a steam chamber at 95°C. The sections were blocked in dPBS with 2.5% normal horse serum for 30 min at room temperature. Sections were stained with the primary antibody, Iba-1 (ionized calcium binding adapter molecule 1, gene symbol AIF1; Fujifilm Wako Pure Chemical Corporation, Osaka, Japan) in blocking buffer (1:1000) and placed on a rotating platform at 4°C overnight. The following day, after washes in dPBS, secondary ImmPress – HRP Horse Anti-Rabbit IgG Polymer (Vector Laboratories, Burlingame, CA) was applied for 1 h at room temperature. Visualization was achieved utilizing Nova Red and counterstained with Hematoxylin QS (both from Vector Laboratories, Burlingame, CA).

*In situ* hybridization for SIV RNA was performed using RNAscope (Advanced Cell Diagnostics, Newark, CA) with probes targeting SIV (which targets SIV coding sequences of gag, pol, vif, vpx, vpr, nef, and tat). Target amplification and color development was done using the 2.5 HD RED Assay (Advanced Cell Diagnostics, Newark, CA) and sections counterstained with Hematoxylin QS following manufacturer’s protocol.

#### NanoString and quantitative real-time PCR (qRT-PCR)

Total RNA was extracted according to manufacturer’s instructions from 70 to 180 mg of the indicated brain regions, liver, and spleen using TRIzol (Life Technologies, Carlsbad, CA). RNA was further purified using the RNA Clean-Up protocol using the Qiagen RNeasy Mini Kit (Qiagen, Germantown, MD). The nCounter NHP Immunology expression panel from NanoString was performed by UNMC Genomics Core using the nCounter digital analyzer 5s DNA RNA. The gene targets and they percent identity with *M. mulatta* are listed in Table S2. Selected transcripts were validated by qRT-PCR. For qRT-PCR, two μg of total RNA was reversely transcribed to a total volume of 100 μL cDNA. Sixteen μL RNA (125 ng/μL), 8 μL random primers (30 μg/μL) and 3 μL dNTPS (10 mM) (Invitrogen, CA), were incubated at 65°C for 10 min and 4°C for 5 min. Four μL 100 μM DTT, 8 μL 25 mM MgCL2, 8 μL 5X First Strand Buffer and 1 μL SUPERase.IN (20 U/μL) (Invitrogen, Carlsbad, CA) were added and incubated at 42°C for 2 min and 4°C for 5 min. Lastly, 1 μL of Invitrogen SuperScript IV Reverse Transcriptase (200 U/μL) (Invitrogen, Carlsbad, CA) was added and the reaction was incubated at 25°C for 10 min, 42°C for 60 min, and 70°C for 15 min with a 4°C infinite hold. Total volume was brought up to 100 μL with RNase free water. Real-Time PCR was performed using the Step-One Plus real-time PCR machine. Each 20 μL reaction contained 1× TaqMan Gene Expression Master Mix (Applied Biosystems, CA), 5 μL cDNA template, and primer probe sets. Primer probe sets were purchased from Thermo Fisher Scientific (Waldham, MA): CXCL11 #Rh02621763_m1, OAZ1 #Rh02809570_gH, OAS2 #Rh02842591_m1, IRF1 #Rh00971962_m1, MX1 #Rh02842279_m1, ALAS1 #Rh02829381_m1. The TBP Primer Probe Set was synthesized by Eurogentec (Freemont, CA): TBP Forward: aaagaccattgcacttcgtg TBP Reverse: ggttcgctctcttatcc and TBP Probe: tcccaagcggtttgctgcag. The cycling profile used for PCR reactions was as follows: 2 min 50°C, 10 min at 95°C, followed by 40 cycles with 15 s at 95°C and 1 min at 60°C. For determination of relative expression, the C_t_ values of ALAS1, OAZ1 and TBP were averaged for housekeeping gene normalization.

### Quantification and statistical analysis

If not otherwise mentioned, statistical analyses were performed using Graph Pad Prism 9.0. Prior to analyses, all assays were analyzed for normality and outliers were removed using Grubb’s test for outliers. Differences between viral loads and immune cell subsets in groups of SIV-infected animals treated with saline or deprenyl were analyzed using multiple T-tests or Mann-Whitney tests if data were non-parametric and correcting for multiple comparisons using the Holm-Sidak method. For peripheral biomarkers, to enable comparisons between analytes in which the mean of one group was below the limit of detection, analyte values were set to the one-half of the limit of detection for the purpose of graphing and analysis. Analytes were determined to be undetectable if less than 5 of the 16 samples contained detectable analyte. Changes in peripheral biomarkers were analyzed using multiple T-tests (Mann-Whitney tests if data were non-parametric) and correcting for multiple comparisons using the Holm-Sidak method.

Concentrations of dopamine, serotonin, and metabolites were analyzed using an ordinary 2-way ANOVA, assessing the effects of deprenyl and SIV infection. For flow cytometry, Graph Pad Prism 9.0 software was used to carryout statistical comparisons and plot figures. Descriptive statistics were presented to summarize continuous variables. Wilcoxon rank sum tests were used to determine the statistically significant difference in continuous outcomes between groups for flow cytometry data. To analyze the gene expression data generated using the NanoString array, NanoString nSolver was used for background thresholding and normalization of gene counts prior to t-tests, with multiple testing correction by the Benjamimi-Yekutieli method. The housekeeping genes used for all analyses were ALAS1, DDX50, ERCC3, HDAC3, OAZ1, PPIA, and TBP. Effects were considered significant if the p value was below 0.05.

## Data Availability

• All data reported in this paper will be shared by the Lead contact upon request. • This paper does not report original code. • Any additional information required to reanalyze the data reported in this paper is available from the Lead contact upon request.

## Supplemental information

Document S1. Figure S1

Table S1. Values of detected analytes in healthy macaques, related to Table 1 UN; undetectable

Table S2. Nanostring genes with % identity to Macaca mulatta, related to Figure 5 % identity to Macaca mulatta refers to the sequence homology between the human genes in the panel and the corresponding Macaca mulatta gene

Table S3. Genes that are significantly altered by deprenyl in SIV-infected animals, related to Figure 5 All genes in this table showed a significant change (>2-fold, corrected p-value <0.05) in response to deprenyl treatment in SIV-infected macaques
