# Supplementary material for: Deprenyl reduces inflammation during acute SIV infection
Source: iScience. 2022 Apr 6;25(5):104207. doi: 10.1016/j.isci.2022.104207 (PMC9046124; doi:10.1016/j.isci.2022.104207)
Supplement: Document S1. Figure S1 [file mmc1.pdf]

iScience, Volume *25*

Supplemental information

Deprenyl reduces inflammation

during acute SIV infection

K.M. Emanuel, K. Runner, Z.D. Brodnik, B.M. Morsey, B.G. Lamberty, H.S. Johnson, A. Acharya, S.N. Byrareddy, R.A. España, H.S. Fox, and P.J. Gaskill

Supplemental Figures and Tables

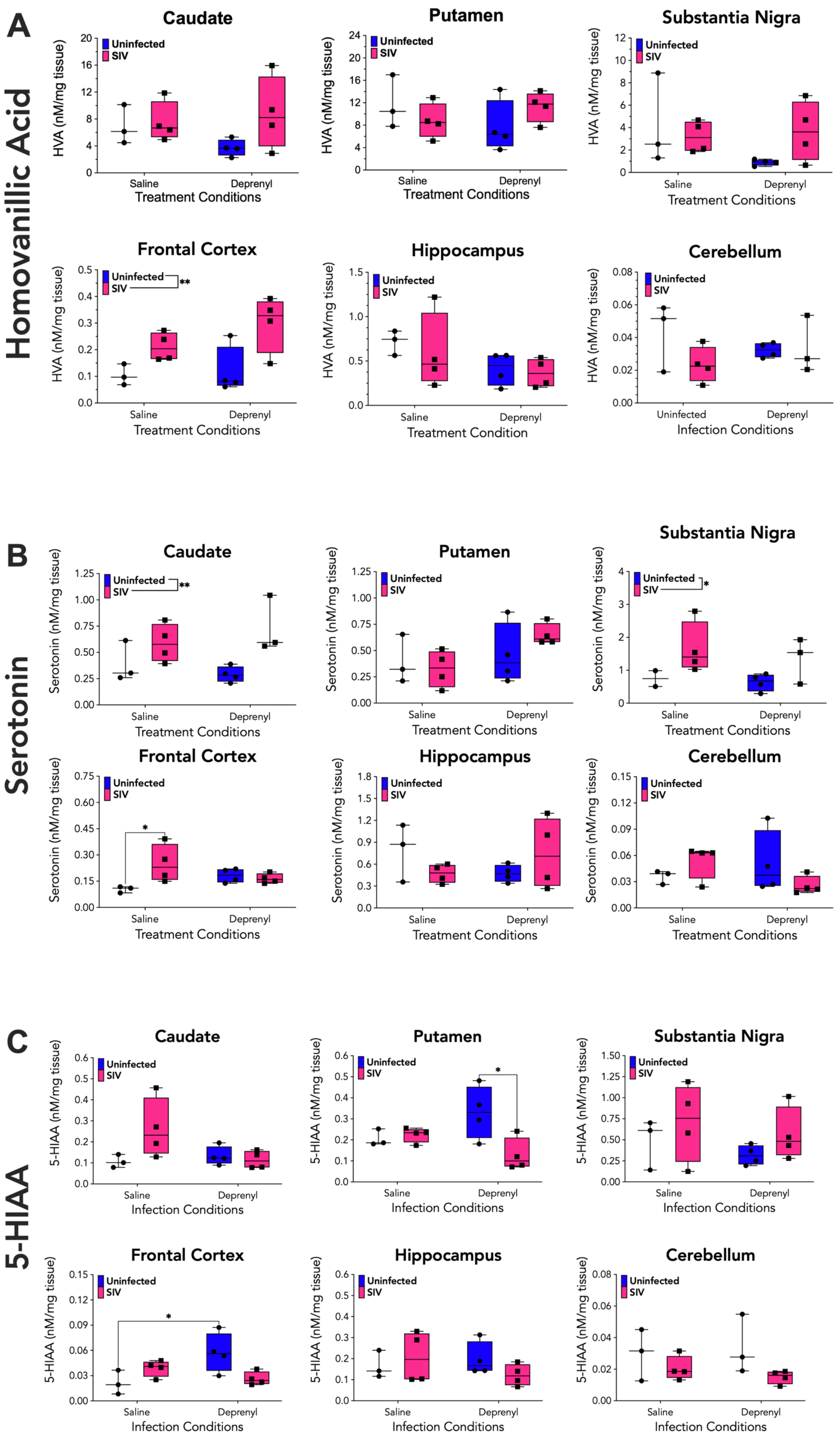

Figure S1, Related to Figure 6. Brain sections were rapidly dissected in ice-cold HeGA to protect monoamines from oxidation then analyzed for monoamines and metabolites by HPLC. Two samples were analyzed from the caudate, putamen, frontal cortex, hippocampus, and cerebellum of all SIV-infected animals (4 SIV + saline and 4 SIV + deprenyl), while a single sample was examined from the substantia nigra. Each brain region was analyzed for the concentrations of the monoamine metabolite homovanillic acid, serotonin (5-HT) and the serotonin metabolite 5-hydroxyindoleacetic acid (5-HIAA). (**A**) Treatment with deprenyl did not significantly change the concentration of HVA in any of the brain regions examined, but SIV infection significantly increased HVA concentrations in the frontal cortex [2-way ANOVA, SIV x deprenyl, frontal cortex; SIV, $F(1,11) = 11.95$, $^{**}p = 0.0054$; deprenyl, $F(1, 11) = 1.528$, $p = 0.2421$]. (**B**) While treatment with deprenyl did not significantly change the concentration of 5-HT in any of the brain regions examined, deprenyl did have a significant interaction with SIV in the frontal cortex, as SIV + saline animals showed a significant increase in serotonin in this region, while this effect was lost in deprenyl treated animals [2-way ANOVA, SIV x deprenyl, frontal cortex; SIV, $F(1,11) = 3.940$, $p = 0.0727$; deprenyl, $F(1, 11) = 0.0099$, $p = 0.9226$; SIV x deprenyl, $F(1,11) = 6.273$, $^{*}p = 0.0293$; Tukey's saline:uninfected vs. saline:SIV $^{*}p = 0.0460$]. Further, SIV infection significantly increased serotonin concentrations in the caudate and substantia nigra [2-way ANOVA, SIV x deprenyl; (caudate; SIV, $F(1,10) = 10.37$, $^{**}p = 0.0092$; deprenyl, $F(1, 10) = 0.0445$, $p = 0.8371$); (substantia nigra; SIV, $F(1,10) = 5.676$, $^{*}p = 0.0411$; deprenyl, $F(1, 10) = 0.081$, $p = 0.7824$). (**C**) Deprenyl showed significant interaction with SIV infection in the putamen, significantly reducing 5-HIAA in SIV infected but not uninfected animals [2-way ANOVA, SIV x deprenyl; putamen; SIV, $F(1,11) = 0.1099$, $p = 0.7464$; deprenyl, $F(1, 11) = 4.772$, $p = 0.0515$; SIV x deprenyl, $F(1,11) = 6.751$, $^{*}p = 0.0248$, Tukey's deprenyl:uninfected vs. deprenyl:SIV $^{*}p = 0.0213$]. There was also a significant interaction between deprenyl and SIV infection in regard to 5-HIAA in the frontal cortex, as deprenyl significantly increased 5-HIAA levels in uninfected but not SIV-infected macaques [2-way ANOVA, SIV x deprenyl; frontal cortex; SIV, $F(1,11) = 2.228$, $p = 0.1636$; deprenyl, $F(1, 11) = 0.7293$, $p = 0.4113$; SIV x deprenyl, $F(1,11) = 9.317$, $^{*}p = 0.0110$; Tukey's saline:uninfected vs. deprenyl:uninfected $^{*}p = 0.0432$]. Further, there was a strong trend toward an SIV x deprenyl interaction in the caudate ($p = 0.0669$) and a strong trend toward SIV alone reducing 5-HIAA in both the putamen ($p = 0.0515$) and cerebellum ($p = 0.0572$).

Table S1. Values of detected analytes in healthy macaques, Related to Table 1

UN; undetectable

Table S2. Nanostring genes with % identity to *Macaca mulatta*, Related to Figure 5

% identity to *Macaca mulatta* refers to the sequence homology between the human genes in the panel and the corresponding *Macaca mulatta* gene

Table S3. Genes that are significantly altered by deprenyl in SIV-infected animals, Related to Figure 5

All genes in this table showed a significant change (>2-fold, corrected p-value <0.05) in response to deprenyl treatment in SIV-infected macaques
